# Extracellular biosynthesis, OVAT/statistical optimization, and characterization of silver nanoparticles (AgNPs) using *Leclercia adecarboxylata* THHM and its antimicrobial activity

**DOI:** 10.1186/s12934-022-01998-9

**Published:** 2022-12-30

**Authors:** Hany M. Abdelmoneim, Tarek H. Taha, Mohamed S. Elnouby, Hala Mohamed AbuShady

**Affiliations:** 1 Microbiology Department, Faculty of Science, Ain Shams University, Cairo, Egypt; 2 Environmental Biotechnology Department, Genetic Engineering and Biotechnology Research Institute (GEBRI), City of Scientific Research and Technological Applications (SRTA-City), New Borg El-Arab City, Alexandria, 21934 Egypt; 3 Composite and Nanostructured Materials Research Department, Advanced Technology and New Materials Research Institute (ATNMRI), City of Scientific Research and Technological Applications (SRTA-City), New Borg El-Arab City, Alexandria, 21934 Egypt

**Keywords:** Biosynthesis of AgNPs, Optimization, TEM, FTIR, Antimicrobial activity, MIC

## Abstract

**Background:**

The biosynthesis of silver nanoparticles (AgNPs) is an area of interest for researchers due to its eco-friendly approach. The use of biological approaches provides a clean and promising alternative process for the synthesis of AgNPs. We used for the first time the supernatant of *Leclercia adecarboxylata* THHM under optimal conditions to produce AgNPs with an acceptable antimicrobial activity against important clinical pathogens.

**Results:**

In this study, soil bacteria from different locations were isolated and screened for their potential to form AgNPs. The selected isolate, which was found to have the ability to biosynthesize AgNPs, was identified by molecular methods as *Leclercia adecarboxylata* THHM and its 16S rRNA gene was deposited in GenBank under the accession number OK605882. Different conditions were screened for the maximum production of AgNPs by the selected bacteria. Five independent variables were investigated through optimizations using one variable at a time (OVAT) and the Plackett–Burman experimental design (PBD). The overall optimal parameters for enhancing the biosynthesis of AgNPs using the supernatant of *Leclercia adecarboxylata* THHM as a novel organism were at an incubation time of 72.0 h, a concentration of 1.5 mM silver nitrate, a temperature of 40.0 °C, a pH of 7.0, and a supernatant concentration of 30% (v/v) under illumination conditions. The biosynthesized AgNPs have been characterized by UV–visible spectroscopy (UV–Vis), transmission electron microscopy (TEM), and Fourier transform infrared spectroscopy (FTIR). The biosynthesized AgNPs showed an absorption peak at 423 nm, spherical shape, and an average particle size of 17.43 nm. FTIR shows the bands at 3321.50, 2160.15, and 1636.33 cm^−1^ corresponding to the binding vibrations of amine, alkyne nitrile, and primary amine bands, respectively. The biosynthesized AgNPs showed antimicrobial activity against a variety of microbial pathogens of medical importance. Using resazurin-based microtiter dilution, the minimum inhibitory concentration (MIC) values for AgNPs were 500 µg/mL for all microbial pathogens except for *Klebsiella pneumoniae* ATCC13883, which has a higher MIC value of 1000 µg/mL.

**Conclusions:**

The obtained data revealed the successful green production of AgNPs using the supernatant of *Leclercia adecarboxylata* THHM that can be effectively used as an antimicrobial agent against most human pathogenic microbes.

## Introduction

Nanotechnology is the science of materials, which involves manipulating matter on a very small scale, with a size in the range of 1–100 nm [1, 2]. Nanotechnology is one of the most vital research fields in materials science focusing on the synthesis and applications of nanoparticles [3]. At the nanoscale level, materials have unique chemical, physical, optical, magnetic, and electrical properties due to their large surface area to volume ratio [3, 4]. The new properties of nanoparticles depend on their size, shape, and morphology, allowing them to interact with plants, animals, and microbes [5]. Metal nanoparticles are important as they have potential applications in catalysis, photonics, biomedicine, antimicrobial activity, and optics [6]. Among all the novel metals, silver has been used as an antimicrobial agent since ancient times [3]. It has gained a lot of attention due to its medicinal, clinical, and culinary properties, with recently observed enormous effectiveness as an anticancer agent [7]. The biosynthesis of nanoparticles, like silver nanoparticles (AgNPs), is an important area of nanotechnology research [8]. However, AgNPs are gaining considerable interest due to their diverse use in various areas such as drug delivery [9], gene delivery systems [10], nanodevice manufacturing, and medicine [11, 12]. In addition, it is used extensively because of the therapeutic perspective, such as antibacterial, antifungal, inflammatory response, antiviral, and anticancer activities [13–16]. Biosynthesis of AgNPs has advantages over physical and chemical synthesis methods as being eco-friendly, feasible, and easily scalable for extensive quantities of synthesis [3, 17]**.** Moreover, biosynthesized AgNPs reduced the time, temperature, size, shape, and toxicity levels [18]. Many scientists have made efforts to make use of various microorganisms, including fungi, bacteria, yeasts, and actinomycetes, which can produce AgNPs by intracellular or extracellular pathways [3]. Numerous scientists have used bacterial strains in the biosynthesis of AgNPs due to their rapid growth rate and highly efficient enzymatic system [19]. The biosynthesis of AgNPs with distinct size and morphology by using bacteria is reported for the first time for the bacterium *Pseudomonas stutzeri* AG 259, which was isolated from a silver mine [20–22]. Extracellular production is more prioritized than intracellular, which requires extraction and purification of AgNPs from the microbial growth [23]. In addition, the extracellular production was confirmed to include high amounts of proteins, which acted as capping agents [24]. One of the mechanistic aspects of AgNPs biosynthesis is the secreted enzymes by bacteria that act as reducing agents for silver ions [25]. AgNPs have a strong bactericidal effect against a broad spectrum of bacteria such as *Pseudomonas* sp., *Escherichia* sp., *Vibrio* sp., and *Salmonella* sp. [26]. Furthermore, the biosynthesized AgNPs showed significant antifungal potential against *Candida tropicalis* and *C. albicans*, as reported by [27]. The goal of our research work was to isolate, screen, and identify the most potent bacteria that reduce silver ions into AgNPs by their aqueous bacterial supernatant. To the best of our knowledge, the present study reports, for the first time, the biosynthesis of AgNPs using *Leclercia adecarboxylata* cultural supernatant. The biosynthesized AgNPs were characterized by different methods, including UV–visible spectroscopy, TEM, and FTIR. In addition, this work will investigate the findings relating to optimization of different experimental parameters, the antimicrobial effect, and the MIC of the biosynthesized AgNPs against clinically microbial pathogens.

## Results and discussion

Nanotechnology is a broad term referred to all advanced technologies involving the nanoscale [28]. There are three different methods for the production of nanoparticles, physical, chemical, and biological methods [29], but the best one, that is nontoxic and eco-friendly, is the biological technique [30]. In the current study, soil bacteria from different locations were isolated and screened for their potential to form AgNPs. The selected isolate, which was found to have the ability to biosynthesize AgNPs, was subjected to identification by molecular methods. The reduction of silver ions was checked by visual inspection as well as by measuring its UV–visible absorption. Further characterization by transmission electron microscopy confirmed the synthesis of stable AgNPs.

### Isolation of soil bacteria

A total of twenty collected soil samples were serially diluted in sterile normal saline solution and were then plated onto nutrient agar plates. The selected colonies were further subcultured on nutrient agar plates. A total of 25 bacterial isolates representing different colony morphologies were isolated from the twenty soil samples and were encoded by the symbols from S1–S25.

### Screening for extracellular production of AgNPs

**Fig. 1:**
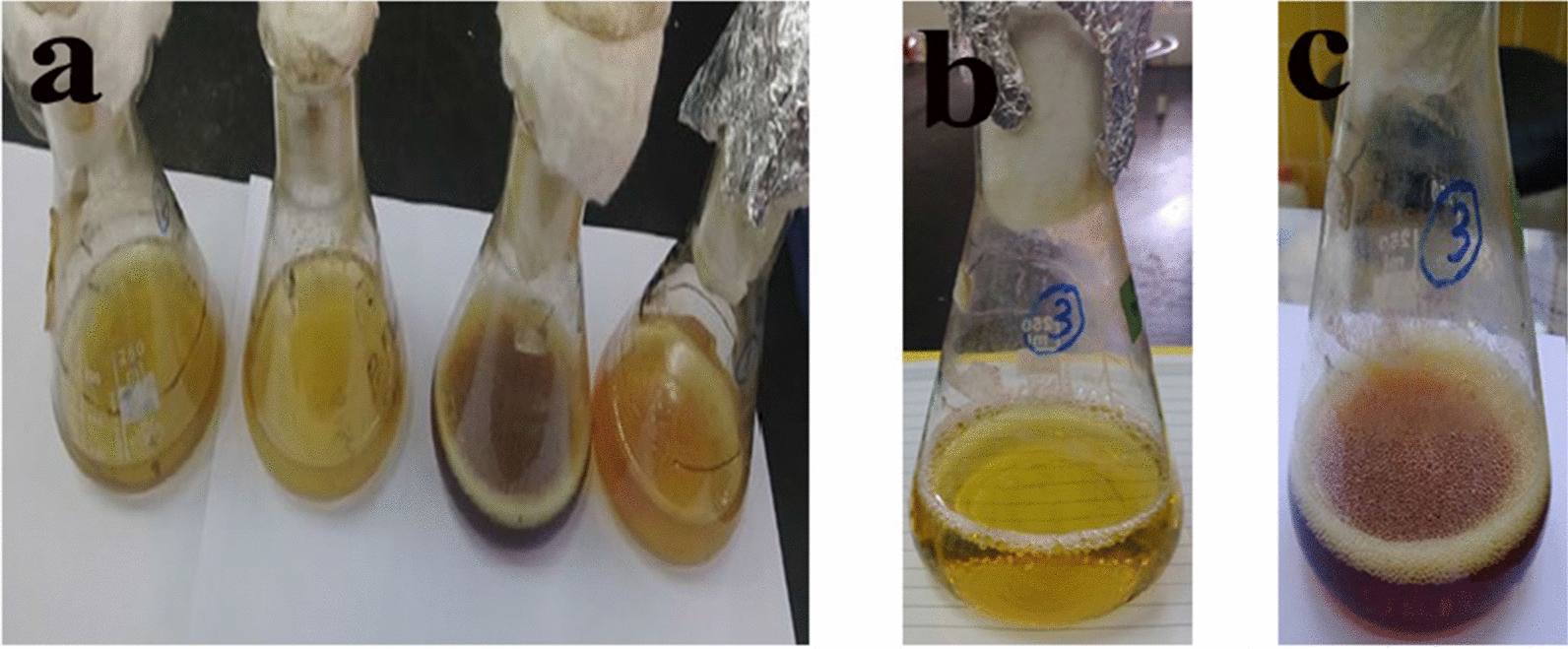
Visual observation of the biosynthesis of silver nanoparticles by bacterial isolates supernatants. **a**: Screening for biosynthesis of silver nanoparticles using various bacterial supernatants. **b**: Control, bacterial supernatant of S3 isolate without AgNO_3_ solution (no color change). **c**: Biosynthesis of AgNPs by S3 supernatant with AgNO_3_ solution (The color changed from yellow to dark brown)

The current study was focused on the extracellular synthesis of AgNPs by bacterial supernatant. A total of 25 isolates that had been previously isolated and purified were checked for their ability to produce AgNPs through the separate inoculation into LB broth that lacks NaCl. The biosynthesis of AgNPs using bacterial supernatants was investigated primarily through the observation of color change of the experimental samples in the presence of 1.0 mM AgNO_3_ final concentration. Observation of color change is a method generally used for screening microbial isolates for silver nanoparticle’s biosynthesis [31–35]. The screening revealed that, among 25 tested isolates, only one isolate number 3 (S3) showed the ability to synthesis AgNPs. After 48 h of incubation of the bacterial supernatants with AgNO_3_ solution, the color of the supernatant of isolate S3 was changed from yellow to dark brown, in contrast to the negative control (Fig. 1). At the same time, the experimental negative control containing supernatant without AgNO_3_ showed no color change. This suggests that the color change observed in the bacterial supernatant sample was due to the formation of AgNPs. The positive result as observed by the formation of brown color was maintained throughout the 168 h period of observation. The obtained results are in agreement with those obtained by Abd-elnaby et al. [36], who reported that the formation of AgNPs by actinomycetes was observed by color change from pale yellow to yellowish-brown. A similar observation was previously reported for the extracellular filtrate of *Bacillus megaterium*, where a pale yellow to brown color was formed due to the reduction of AgNO_3_ solution to AgNPs [37]. Similarly, Nayaka et al. [38] reported that a change in the color from pale yellow to brown after incubation of the culture supernatant of *Streptomyces* sp. NS-33 with AgNO_3_ was obtained by the synthesis of AgNPs.

**Fig. 2:**
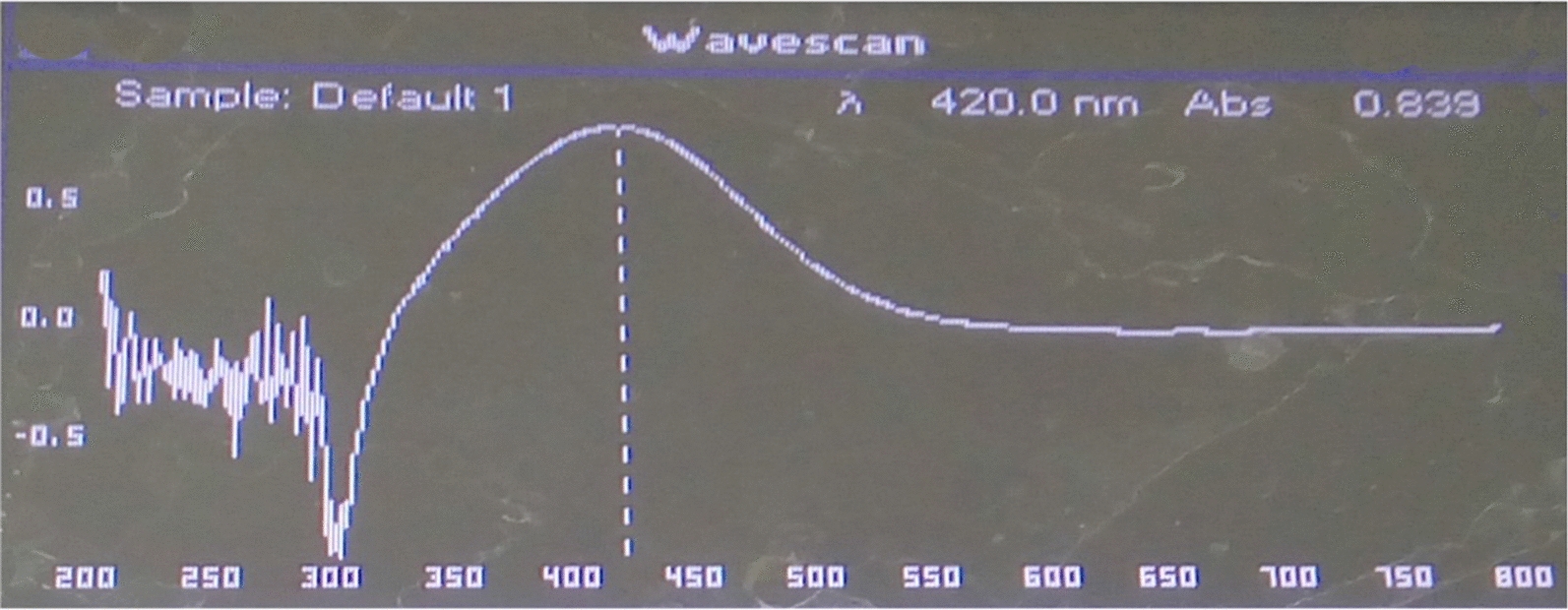
The UV–visible spectra of AgNPs biosynthesized by S3 bacterial supernatant. The absorption of AgNPs was recorded after 48 h of incubation following the addition of 1 mM AgNO_3_ to the bacterial supernatant. The absorption spectrum of AgNPs exhibited a strong peak at 420 nm

The color change from yellow to dark brown observed for the supernatant of isolate S3 was further confirmed by UV–vis spectral analysis as a part of the primary confirmation. The UV–vis absorption spectrum in the range of 200–800 nm of the supernatant of isolate S3 that was changed from yellow to dark brown color is illustrated in Fig. 2. The obtained absorption indicated a strong SPR band maximum at 420 nm, a characteristic peak for AgNPs, which confirms the formation of AgNPs. Methods based on UV–vis spectroscopy have been shown to be an effective technique for the analysis of nanoparticles [35, 39]. The isolate S3 was selected as the successful candidate for the synthesis of AgNPs and was used throughout the rest of the work after its submission for molecular identification.

### Molecular identification of the bacterial isolate

**Fig. 3:**
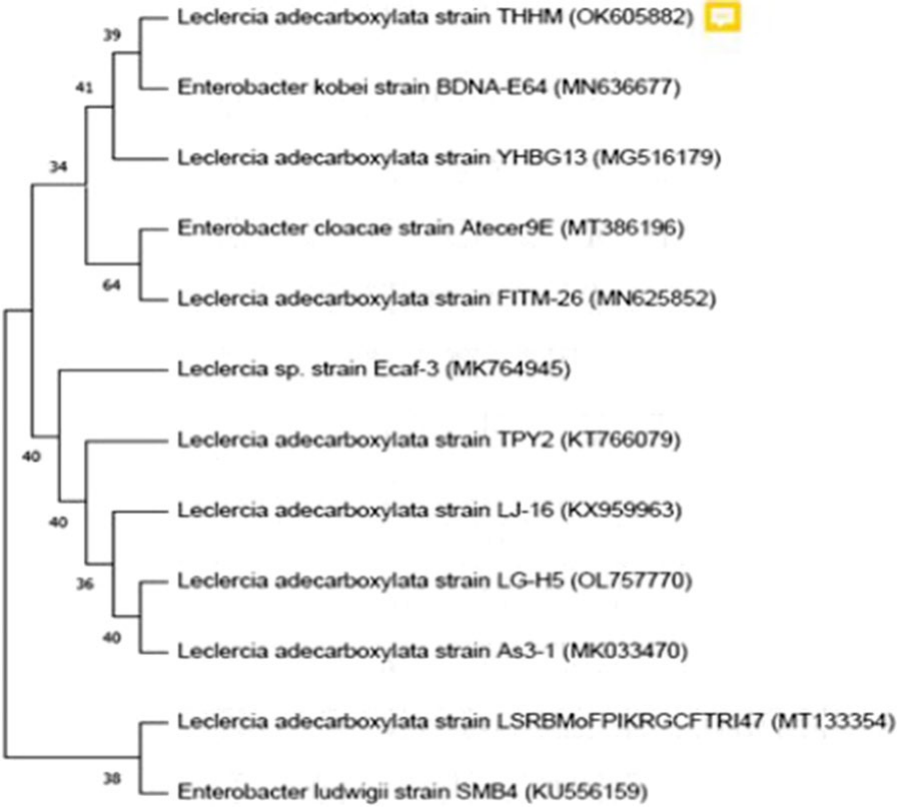
The phylogenetic analysis of the 16S rRNA sequence of the bacterial isolate S3 with other selected sequences from the database. The analysis was conducted using the neighbor-joining method in MEGA 11.0 program. Among the various species of *Leclercia*, the bacterial isolate S3 indicates high similarity to *Leclercia adecarboxylata*

The most potent isolate, S3, that produced silver nanoparticles, was further subjected to molecular identification by a 16S rRNA sequencing-based method. Nowadays, the 16S rRNA gene sequencing of microorganisms has become a useful method for the identification and classification of microorganisms up to the species level [38]. The main goal of this experiment was to verify the bacterial isolate based on genotypic traits. The PCR analysis revealed that the primers succeeded in amplifying the targeted gene with the proposed specific length. The 16S rRNA PCR product was purified and sequenced to obtain the identity of the isolated strain. The obtained 16S rRNA partial sequence was 1282 bp in length. The sequence data were subjected to BLAST analysis, and the results revealed a maximum identity of 99% to other *Leclercia* spp. genes deposited in GenBank. Accordingly, our isolate was named as *Leclercia adecarboxylata* THHM and its sequenced gene was subsequently submitted in the GenBank with the accession number OK605882. The phylogenetic tree of *Leclercia adecarboxylata* THHM with other revealed strains according to the similarity of their sequences is shown in Fig. 3.

*Leclercia adecarboxylata* is a motile Gram-negative bacillus that was first described by Leclerc in 1962 as *Escherichia adecarboxylata* [40], but was reclassified as *Leclercia adecarboxylata* after further studies showed that it belonged to a different genus of the family Enterobacteriaceae [41–43]. Numerous scientists have used bacterial strains in AgNPs biosynthesis due to their unique ability to reduce metallic ions into nanoparticles. To the best of our knowledge, the present study reports, for the first time, the biosynthesis of AgNPs using *Leclercia adecarboxylata* cultural supernatant.

### Optimization of the biosynthesized AgNPs

The optimization of the physicochemical parameters plays a very important role in the production of nanoparticles at a higher rate with better physical, morphological, and biochemical attributes [44]. Different parameters have been optimized for the biosynthesis of AgNPs, including contact time, silver nitrate concentration, pH, temperature, and bacterial supernatant concentration.

### Effect of the incubation periods

**Fig. 4:**
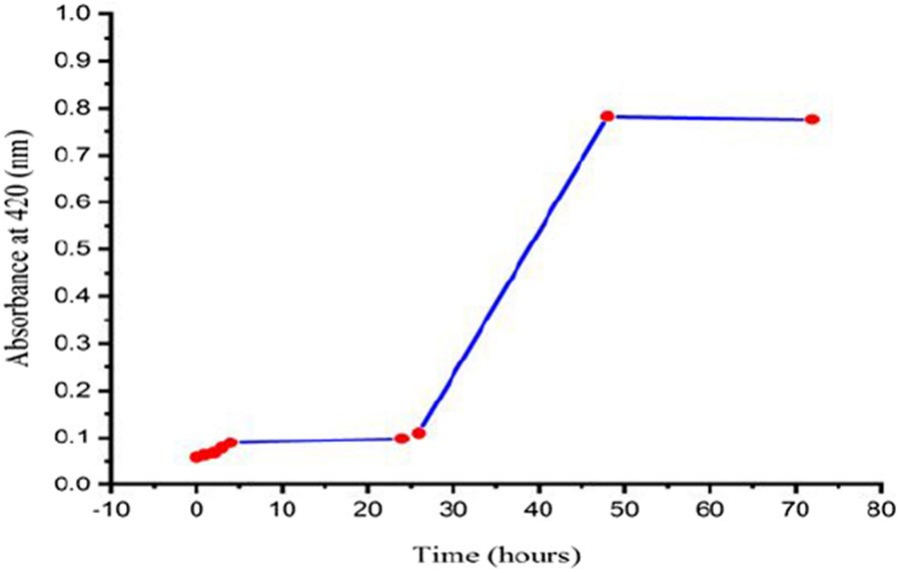
Effect of time duration on the biosynthesis of AgNPs using the supernatant of *Leclercia adecarboxylata* THHM

The incubation period was the first factor to consider for optimizing the biosynthesis of AgNPs. The incubation time is a vital parameter to steer the reaction conditions to tailor the size and shape of the nanostructures [44]. At different time intervals of 0.0, 1.0, 2.0, 4.0, 8.0, 12.0, 24.0, 48.0, and 72.0 h; the biosynthesis of AgNPs using *Leclercia adecarboxylata* THHM supernatant was monitored. The time-dependent extracellular biosynthesis of AgNPs using a 1.0 mM AgNO_3_ final concentration solution is shown in Fig. 4, and represents the plot of the absorbance at 420 nm at different time intervals of the reaction. It was found that no considerable change in the color of the reaction mixture was seen when the incubation time was less than 24.0 h, indicating that no AgNPs were produced, because the redox potential of the silver nitrate was reduced [45]. Furthermore, it was observed that by increasing the time of the reaction, the color of the reaction mixture was changed and the absorption was increased, which means more AgNPs were formed until 48.0 h, but did not increase thereafter, which indicates the stability of the AgNPs colloidal solution [46]. Due to the highest intensity of the measured AgNPs was observed at 48.0 h; this time was considered the optimum time for the current OVAT optimization. In accordance with our findings, similar results were reported by Thamilselvi and Radha [47], who showed that the maximum biosynthesis of AgNPs using *Pseudomonas putida* NCIM 2650 occurred at 48.0 h of incubation.

At the same time, our results are also in agreement with those reported by El-Saadony et al. [45], who mentioned that the maximum biosynthesis of AgNPs was achieved at an incubation time of 40 h using the supernatant of *Bacillus pseudomycoides* MT32, whereas by increasing the incubation time to more than 40.0 h, the biosynthesis of AgNPs did not increase significantly. Other researchers have recorded other incubation times for the biosynthesis of AgNPs, such as 72.0 h [48], 30.0 min [49], and 12.0 min [50].

### Effect of silver nitrate concentration

**Fig. 5:**
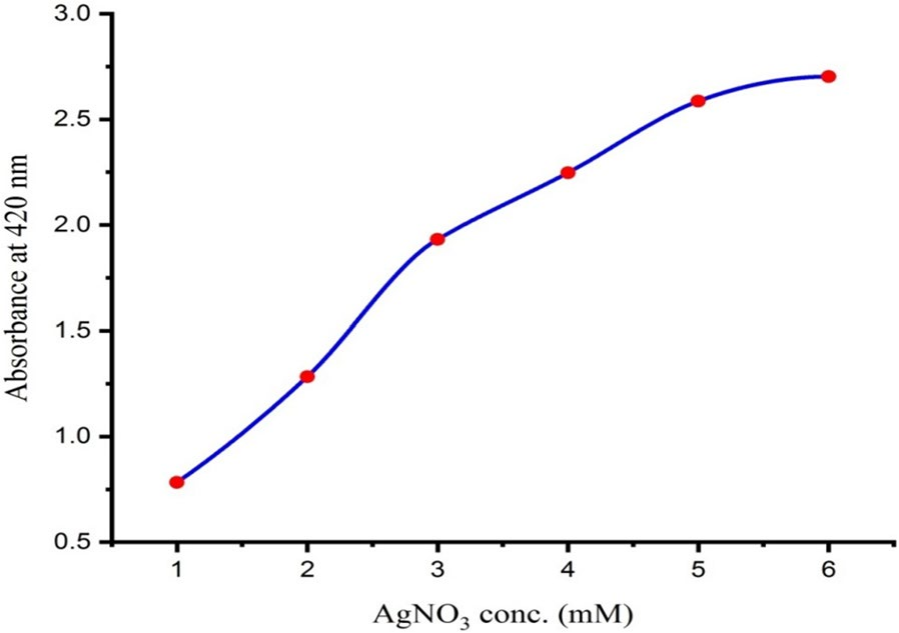
Effect of different AgNO_3_ concentrations on the biosynthesis of AgNPs using the supernatant of *Leclercia adecarboxylata* THHM

To obtain the optimum concentration of AgNO_3_ that yields the maximum and stable production of AgNPs; AgNO_3_ at different concentrations (1.0, 2.0, 3.0, 4.0, 5.0, and 6.0 mM) was added separately to the *Leclercia adecarboxylata* THHM supernatant. The absorbance of the resulting colloidal AgNPs solution was monitored spectrophotometrically. As illustrated in Fig. 5, the absorption spectra showed that by increasing the concentration of the silver nitrate solution, the absorbance of the resulting AgNPs solution was increased. This was reflected by an increase in the measured absorbance at 420 nm. The maximum yield of AgNPs was obtained when the concentration of the AgNO_3_ solution was 6.0 mM, indicating that the rate of bio-reduction is directly proportional to the AgNO_3_ substrate concentration [51]. These results matched those obtained by Nindawat and Agrawal [50], who found that increasing the concentration of AgNO_3_ from 0.5 to 5 mM resulted in an increase in the absorbance during the study of the effect of the AgNO_3_ concentrations on the biosynthesis of AgNPs. In accordance with our findings, similar results were reported by Dwivedi and Gopal [52], who observed that the absorbance peak increased with an increase in the silver nitrate concentration from 0.1 to 5.0 mM, using *Chenopodium album* leaf extract. Our results were correlated with the findings obtained by Saxena et al. [53], who reported that the maximum production of AgNPs by the extracellular filtrate of *Sclerotinia sclerotiorum* MTCC 8785 was at 2.0 mM AgNO_3._ The biosynthesis of AgNPs from bacterial metabolites was found to be better at 1.0 mM AgNO_3_ concentration [54]. Moreover, according to a previous study, the optimal concentration of silver ions in most cases of AgNPs biosynthesis is 1.0 mM [55, 56]. When the concentration of AgNO_3_ exceeds this limit, the amount of AgNO_3_ is not completely reduced. The most stable synthesis of AgNPs was found at an AgNO_3_ concentration of 1.0 mM with no agglomeration for a longer period of time [57]. There was an increase in AgNPs aggregation with an increase in metal ion concentration [58]. Based on the results cited above from many previous researchers [50, 52, 54, 56–59], they observed an increase in silver nanoparticle size, aggregation, and chemical instability as the concentration of silver nitrate ions was increased. Therefore, in the current optimization, a silver nitrate concentration of 1.0 mM was chosen as the recommended concentration for the biosynthesis of AgNPs.

### Effect of pH

**Fig. 6:**
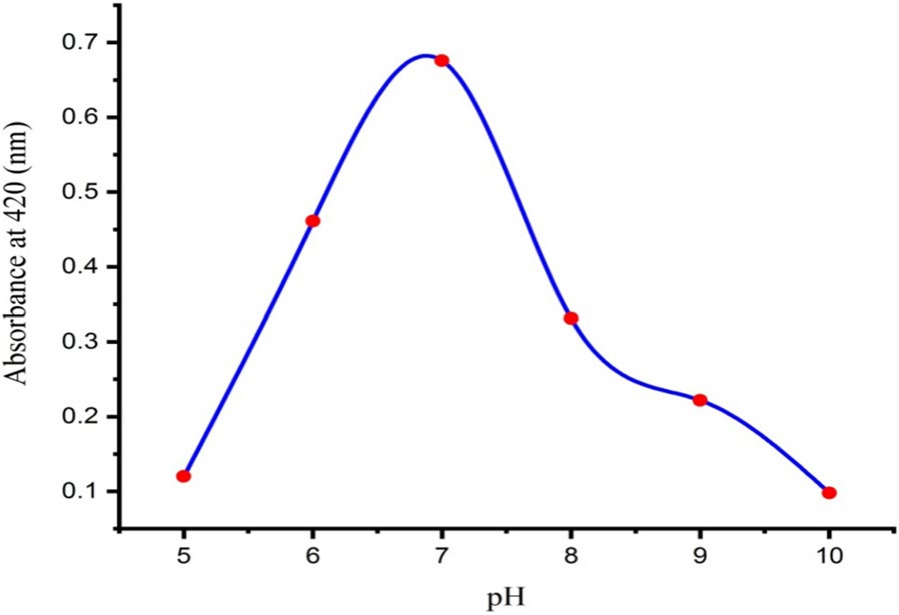
Effect of different pH values on the biosynthesis of AgNPs using the supernatant *Leclercia adecarboxylata* THHM

pH is one of the key factors that plays a major role in the biosynthesis of nanoparticles [60, 61]. The effect of varying pH values on the maximum and stable production of AgNPs was monitored by spectrophotometric analysis. It has been documented that changes in pH influence the shape and size of the nanoparticles, as pH has the ability to alter the charge of biomolecules, which might affect their capping as well as stabilizing abilities [62–64]. Optimization studies with respect to pH revealed that the maximum production of AgNPs occurred at pH 7.0. The results indicated that neutral medium was more suitable for the biosynthesis of AgNPs than acidic or alkaline medium, as the rate of silver ion reduction was higher at pH 7.0 when compared to other pH values, as illustrated in Fig. 6. This indicates that the most favorable pH for the biosynthesis of AgNPs using the supernatant of *Leclercia adecarboxylata* found at pH 7.0 could have been due to the metabolites secreted in the supernatant and capping the nanoparticles. The best conditional pH value was 7.0, which resulted in regular and stable biosynthesis of AgNPs [65]. On the contrary, the acidic and basic pH values reduce the reduction of silver ions into AgNPs. When the pH decreased or increased to 5.0 or 10.0, respectively, there was no production of AgNPs. At low pH, the protein structure is affected and the protein gets denatured and loses its activity, resulting in aggregation of the nanoparticles [59, 66]. Our results were in agreement with those previously reported by Sarsar et al. [67], who found that a sharp peak at pH 7.0 was observed during the biosynthesis of AgNPs. Similarly, El-Dein et al. [23] reported that stable and monodispersed AgNPs were synthesized at pH 7.0 using the supernatant of *Escherichia coli* D8. Other researchers reported that acidic conditions [68, 69] or alkaline conditions [70] are the optimum conditions for biosynthesis of AgNPs.

### Effect of temperature

**Fig. 7:**
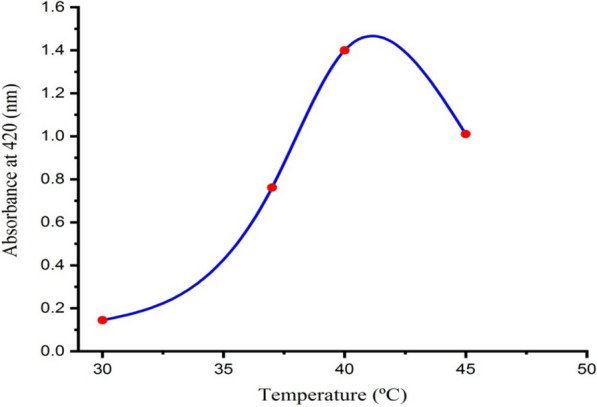
Effect of temperature on the biosynthesis of AgNPs using the supernatant of *Leclercia adecarboxylata* THHM

Temperature is one of the important factors in any chemical and biological reaction as it affects the rate of reaction and plays a major role in controlling the nucleation process during the biosynthesis of AgNPs [1]. The supernatant of *Leclercia adecarboxylata* THHM containing 1.0 mM AgNO_3_ was incubated separately at (30, 37, 40, and 45 ºC) to evaluate the effect of temperature on the biosynthesis of AgNPs. The production of AgNPs was monitored spectrophotometrically as shown in Fig. 7. The results revealed that as the temperature of the reaction mixture increased; the rate of the biosynthesis of AgNPs increased to reach the maximum production at 40 ºC, and thereafter decreased at higher temperatures. This temperature was considered the optimum temperature for the current study. The temperature below and above this temperature did not favor the synthesis of AgNPs. The results showed that with a continuous increase in temperature, the production of AgNPs decreased, and this reduction could be due to the inactivation or degradation of biomolecules responsible for the biosynthesis process [45]. Our findings were in complete correlation with Khan and Jameel [71], who reported that the maximum extracellular biosynthesis of AgNPs mediated by the supernatant of *Fusarium oxysporum* occurred at 40 °C. Our findings were also in agreement with those previously reported by Mittal et al. [72], who found that the absorbance of the biosynthesis of AgNPs reaction mixture increased with increasing temperature from 25 to 45 °C and thereafter decreased at higher temperatures. On the other hand, many studies on the effects of temperature on the biosynthesis of AgNPs were conducted for optimum temperature values and reported different optimum temperatures of 48.5 °C, 25 °C, and 60 °C, using *Bacillus cereus* [73], *Bacillus stearothermophilus* [48], and the cellular extract of *Penicillium oxalicum* GRS-1 [74], respectively.

### Effect of the different supernatant concentrations of the bacterial isolate

**Fig. 8:**
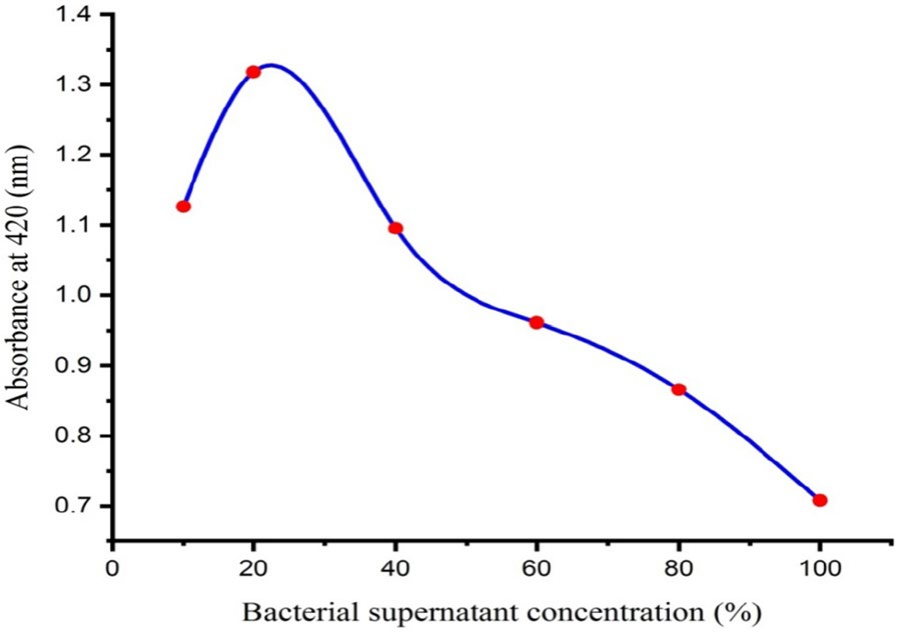
Effect of bacterial supernatant concentration on the biosynthesis of AgNPs using the supernatant of *Leclercia adecarboxylata* THHM bacterial strain

Large numbers of microbes have been found to be capable of biosynthesizing nanoparticles as their cellular extracts act as both reducing as well as capping agents [74]. The effect of different concentrations (10, 20, 40, 60, 80, and 100% (v/v)) of the supernatant of the bacterial isolate was investigated, and the optimal concentration for the maximum production of AgNPs was chosen. It has been shown that the production of AgNPs increased as the supernatant concentration of *Leclercia adecarboxylata* THHM was increased from 10 to 20%. A further increase in the bacterial supernatant concentration of greater than 20% caused a decrease in the biosynthesis of AgNPs. As shown in Fig. 8, among the different tested supernatant concentrations of *Leclercia adecarboxylata* THHM, the 20% supernatant concentration was shown to be the most favorable concentration for the biosynthesis of AgNPs. Increasing the concentration of the bacterial supernatant results in high localized amounts of reducing agents, which leads to the formation of larger clusters or macromolecules and thus the agglomeration of AgNPs [75]. As a result, the 20% supernatant concentration of the *Leclercia adecarboxylata* THHM was chosen as the optimal supernatant concentration for the biosynthesis of the AgNPs in the current optimization. The exact mechanism beyond the supernatant bacterial culture mediated biosynthesis of AgNPs is poorly understood [76]. However, reports suggest that extracellular bio-molecules like enzymes, proteins, amino acids, and carbohydrates secreted by bacteria in their culture supernatant play an important role in the reduction of Ag^+^ ions to AgNPs and their subsequent stabilization by capping [77–79]. The mechanism widely acknowledged is the presence of a nitrate reductase enzyme in the microbial metabolites, which serves as a reducing agent in the biosynthesis of AgNPs [31, 74, 80]. A bacterium belonging to *Leclercia adecarboxylata* was capable of producing the enzyme nitrate reductase and efficient N removal [81]. In agreement with our results, Aboelfetoh et al. [82] found that the activity of the biosynthesis of AgNPs increased with increasing the concentration of *Caulerpa serrulata* extract from 5 to 20%, and a further increase in the extract concentration reduced the biosynthesizing activity. Also, our results were correlated with those Balakumaran et al. [56], who reported that among the different concentrations of fungal biomass tested, the 10% (w/v) biomass of the fungus *Aspergillus terreus* supported the better biosynthesis of AgNPs.

### Optimization of the biosynthesis of AgNPs using Plackett–Burman design (PBD)

Plackett–Burman factorial designs (PBD) is very simple and requires less time when compared with other statistical methods used in experimental design [83]. PBD experiments were effective in determining variables that significantly affected the biosynthesis of AgNPs [84]. This design was used to define the most optimal levels of factors affecting the biosynthesis of AgNPs [35] using the supernatant of *Leclercia adecarboxylata* THHM. In general, this design is a two-factorial one, which identifies the critical parameters required for the elevated production of AgNPs by screening n variables in an n + 1 experiment. The biosynthesis of AgNPs was determined by measuring the spectrophotometric absorbance (as a response) of the resulting solution at 420 nm. Table 1 shows the predicted and actual values of the biosynthesis of AgNPs along with eight different combinations of experimental factors. Each row in the table represents an experiment involving all the five independent variables. The relationship between a set of independent variables and the response was determined by a mathematical model called a multiple regression model using Microsoft Excel 2007 to estimate *t*-value, *P*-value, and confidence level. The analysis of the regression coefficients and *t*-values of five variables is presented in Table 2. In the current study, a total of five independent variables were screened in eight runs using the PBD. The PBD results in Table 1 showed a markedly wide variation in the production of AgNPs in the eight trials in the absorbance range of 0.079–2.199 at 420 nm. This variation reflected the importance of factor optimization to increase the production of AgNPs. The maximum absorbance (2.199) was achieved in the run number 2, while the minimum absorbance (0.079) was observed in the run number 8. The coefficients of each variable represent the level of effect, either positive or negative, of these variables on the biosynthesis of AgNPs. When the sign of the effect of the tested variable is positive, the biosynthesis of AgNPs is greater at a high level of the variable.

**Table 1:** Plackett–Burman design matrix represents the coded values of five independent variables and the absorbance readings at 420 nm as a response reflecting the AgNPs concentration

Trials	A	B	C	D	E	Responses	
						Actual^*^	Predicted
1	+ 1	−1	−1	+ 1	−1	0.442	0.434
2	+ 1	+ 1	−1	−1	+ 1	2.199	2.081
3	+ 1	+ 1	+ 1	−1	−1	1.098	1.216
4	−1	+ 1	+ 1	+ 1	−1	0.302	0.184
5	+ 1	−1	+ 1	+ 1	+ 1	1.523	1.531
6	−1	+ 1	−1	+ 1	+ 1	0.931	1.049
7	−1	−1	+ 1	−1	+ 1	1.192	1.184
8	−1	−1	−1	−1	−1	0.079	0.087

Where the five variables (A-E) are, in order, AgNO_3_ concentration, bacterial supernatant concentration, time, pH, and illumination, respectively. For each variable, −1 represents the low concentration level and + 1 represents the high concentration level

*The experimental values were the mean absorption replicates at 420 nm

**Table 2:** Regression statistical analyses of the Plackett–Burman experimental results

Variable	Coefficient	Standard error	Main effect	Main effect (%)	*t*-Stat	*P*-value	Lower 95.0%	Upper 95.0%
Intercept	0.9708	0.0591	–	–	16.416	0.0037	0.7163	1.2252
AgNO_3_ conc	0.3448	0.0591	0.6895	39	5.8298	0.0282	0.0903	0.5992
Bacterial supernatant conc	0.1618	0.0591	0.3235	18	2.7352	0.1117	−0.0927	0.4162
Time	0.0580	0.0591	0.116	7	0.9808	0.4301	−0.1964	0.3124
pH	−0.1713	0.0591	−0.3425	−19	−2.8959	0.1014	−0.4257	0.0832
Illumination	0.4905	0.0591	0.981	56	8.2945	0.0142	0.2361	0.7449

Analysis of variance (ANOVA)						
	df	SS	MS	*F*-test	Significance *F* (*P*-value)	
Regression	5	3.3464	0.6693	23.923	0.0406	Significant
Residual	2	0.0559	0.0280			
Total	7	3.4023				

Where *t* Student’s test, *P* corresponding level of significance, df degree of freedom, *SS* sum of squares, *MS* mean sum of squares, *F* Fisher’s function; and significance. *F* corresponding level of significance. Multiple R: 0.9917. R square: 0.9835. Adjusted R square: 0.9424

**Fig. 9:**
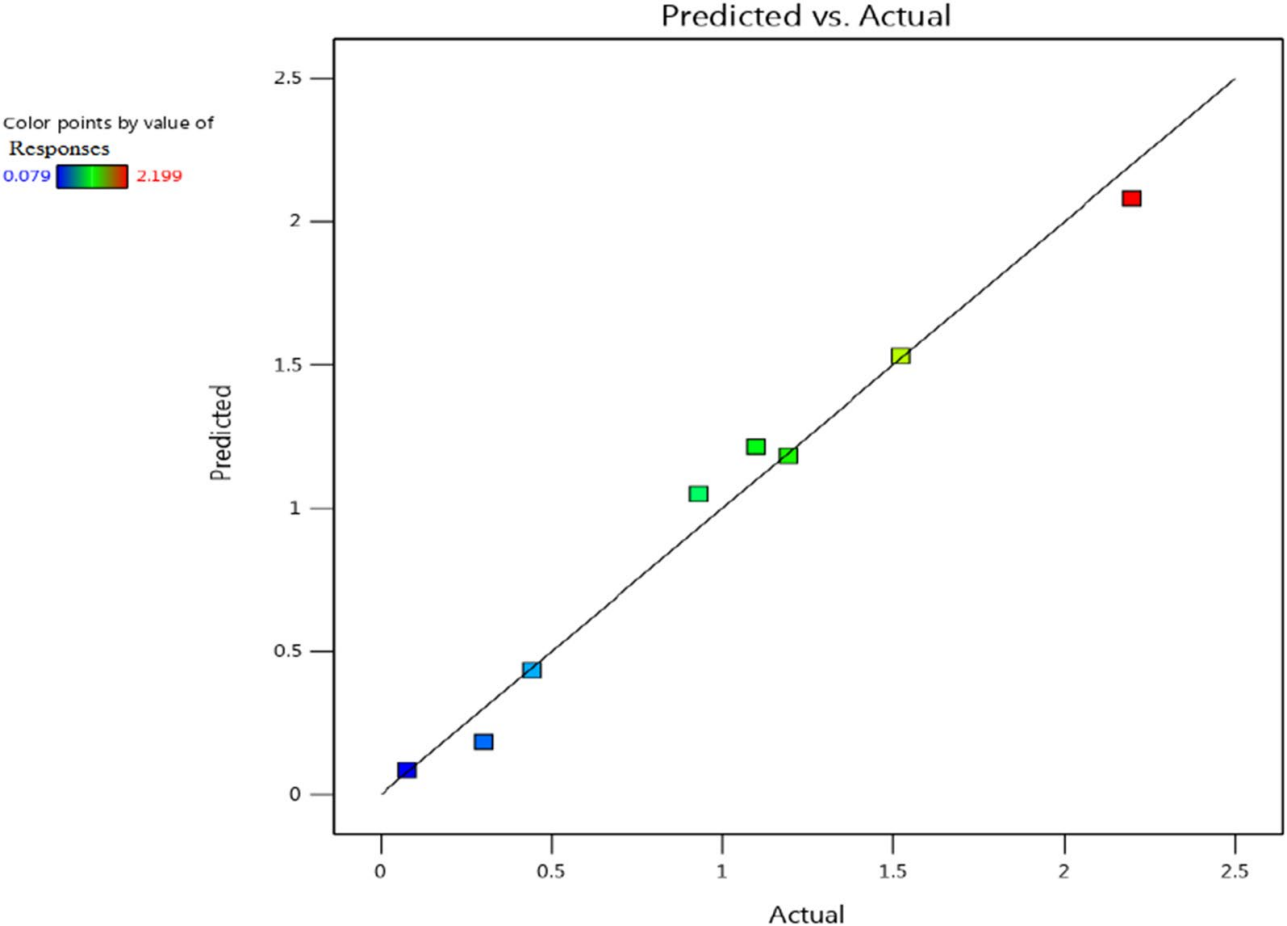
The parity plot implies the correlation between actual/experimental and predicted values of the biosynthesis of AgNPs using the supernatant of *Leclercia adecarboxylata* THHM

**Fig. 10:**
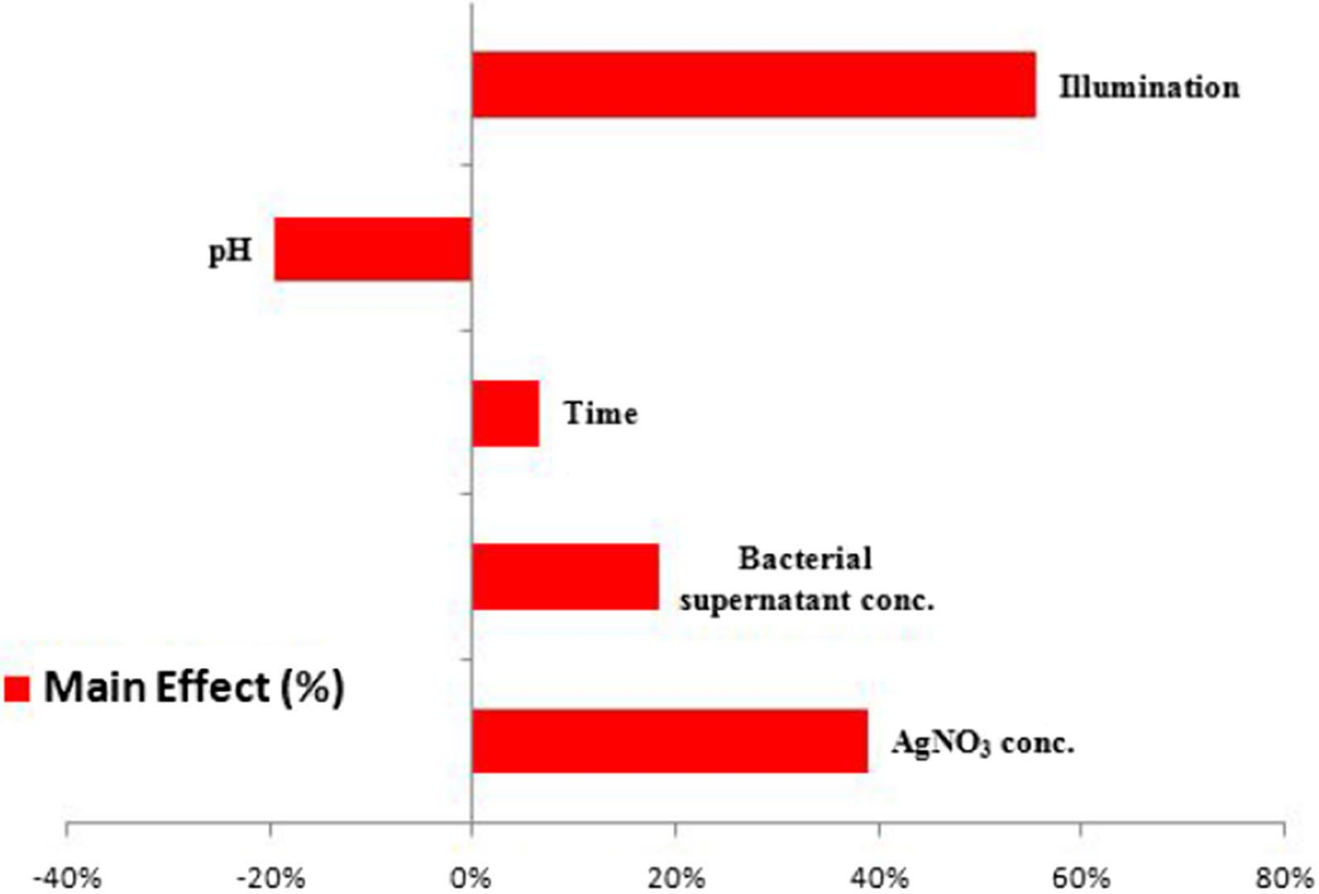
The main effects of the process variables on the biosynthesis of AgNPs using the supernatant of *Leclercia adecarboxylata* THHM according to the Plackett–Burman experimental results

**Fig. 11:**
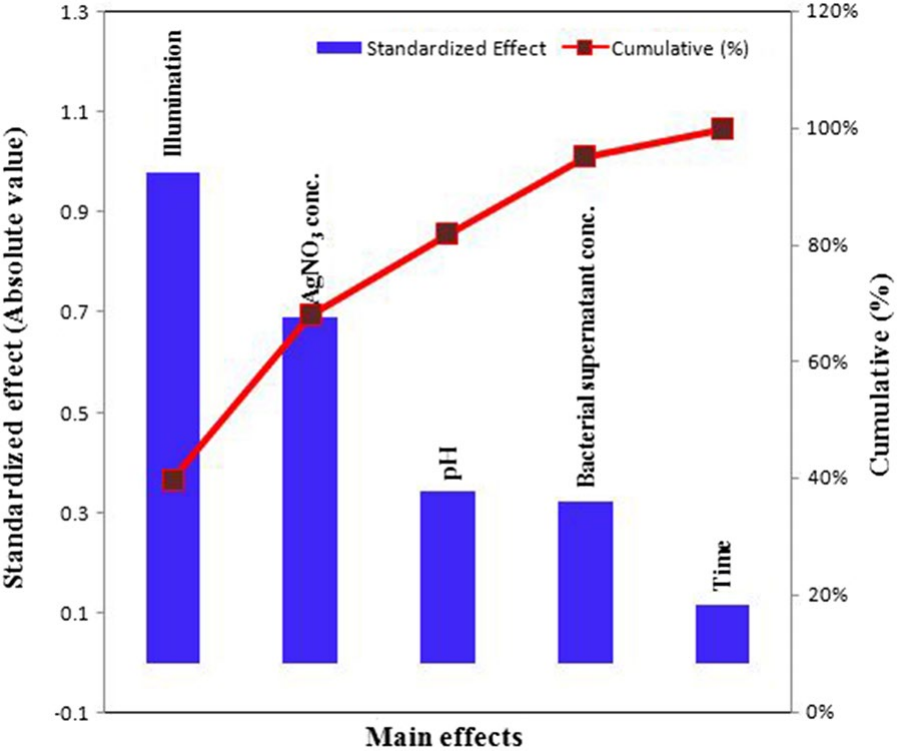
Pareto chart showing the amount of influence of each factor on the biosynthesis of AgNPs

When the sign is negative, the biosynthesis of AgNPs is greater at a low level of the variable. On the other hand, if an effect is close to zero, it means that a factor has little or no effect [83, 85]. The main effects of each variable on the biosynthesis of AgNPs were calculated. The *t*-values for each variable were estimated to identify the statistical significance of the measured response and determine the main effects for the biosynthesis of AgNPs using the supernatant of *Leclercia adecarboxylata* THHM. The coefficient of determination of *R*^2^ was found to be 0.9835, implying that the 98.35% sample variance for the biosynthesis of AgNPs was attributed to the independent variables. This value provides a measure of how much of the variability in the observed response values can be explained by the analysis. The coefficient of determination of (adjusted *R*^*2*^) was calculated to be 0.9424, indicating a good agreement between the experimental and predicted values of the biosynthesis of AgNPs. Figure 9 represents the relationship between the values of the actual biosynthesis of AgNPs using the supernatant of *Leclercia adecarboxylata* THHM and the statistically predicted values. The parity chart indicated a strong correlation between the actual and the statistically predicted values of the biosynthesis of AgNPs. The variables with points near the diagonal line through zero were not significant, while those deviating from the straight line were significant [86]. With respect to the main effect of each variable (Fig. 10), among the five variables, illumination, AgNO_3_ concentration, bacterial supernatant concentration, and time showed a positive sign of the effect on the biosynthesis of AgNPs, while only pH showed a negative sign of the effect. Based on the present results, higher levels of illumination, AgNO_3_ concentration, bacterial supernatant concentration, and time, respectively, and the low level of pH can increase the biosynthesis of the AgNPs using the supernatant of *Leclercia adecarboxylata* THHM. As a result, the optimal parameters for improving the biosynthesis of AgNPs and will be used in the final production of AgNPs were at an incubation time of 72.0 h, a concentration of 1.5 mM silver nitrate, a pH of 7.0, and a supernatant concentration of 30% (v/v) under illumination conditions at a temperature of 40.0 °C. The analysis of the results yielded the standardized Pareto chart of the main effects, which showed the order of the effects on the biosynthesis of AgNPs in Fig. 11. The Pareto chart showed that illumination, AgNO_3_ concentration, and pH are the most important significant factors influencing the biosynthesis of AgNPs. Time was the most insignificant factor. Both of the statistical parameters, *t*-value and *P*-value, were used to confirm the significance of the factors studied. The model *F* value of 23.923 (Table 2) indicates that the model was significant. The values of significance *P* < 0.05 (0.0406) indicate that the model terms were significant with confidence levels greater than 95%. In a similar study conducted by Trivedi et al. [87], six different variables were screened with the PBD to investigate the effect of variables on the biosynthesis of AgNPs using citrus peel extract. Temperature, pH, volume of reductant, volume of reaction vessel, illumination, and silver nitrate concentration were the variables chosen. They reported that temperature was the most significant factor affecting the biosynthesis of AgNPs, followed by illumination and pH, while the concentration of silver nitrate was the least significant factor. Inparallel to our findings, Halima et al. [88] reported that optimization of nine factors influencing the biosynthesis of AgNPs from leaf extract of *Piper betel* and *Jatropha curcas* using PBD appeared that plant extract, silver nitrate concentration, and sunlight has the greater influence on the biosynthesis of the AgNPs with significant *F* and *P*-values.

### Characterization of the synthesized AgNPs

**Fig. 12:**
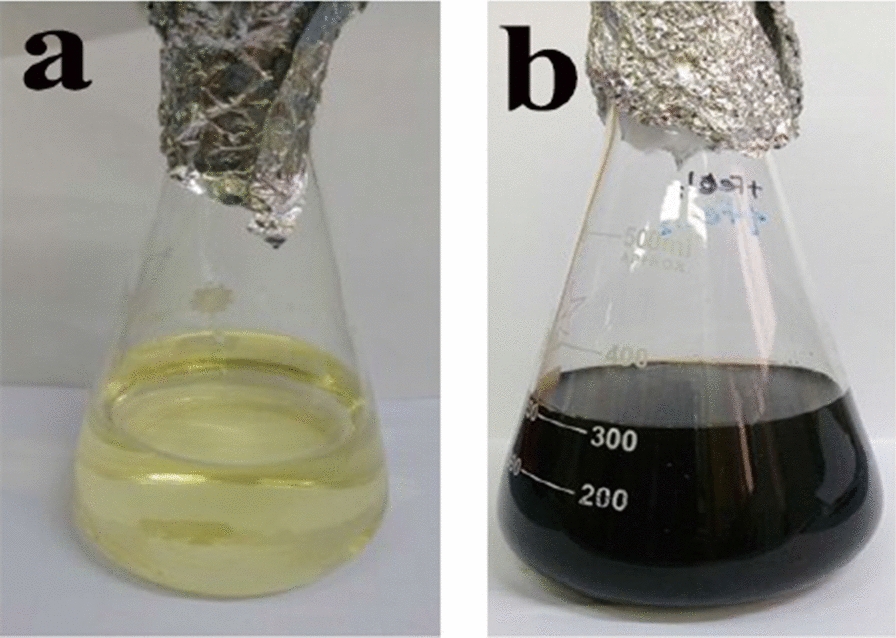
Visual observation of the bio-AgNPs using the supernatant of *Leclercia adecarboxylata* THHM under optimized conditions. **a** Bacterial supernatant in the absence of an AgNO_3_ solution (no color change). **b** Biosynthesized AgNPs (the color has changed from yellow to dark brown)

For the characterization purpose of biosynthesized AgNPs (bio-AgNPs), several analytical techniques were employed, including UV–visible spectroscopy, TEM, and FTIR [89, 90]. Metallic nanoparticles experience color changes depending on their size in the nanoscale region [91]. Visual observation of the supernatant of *Leclercia adecarboxylata* THHM incubated with AgNO_3_ under optimized conditions showed a color change from yellow to dark brown of the biosynthesized AgNPs (Fig. 12).

### UV–vis spectroscopy

**Fig. 13:**
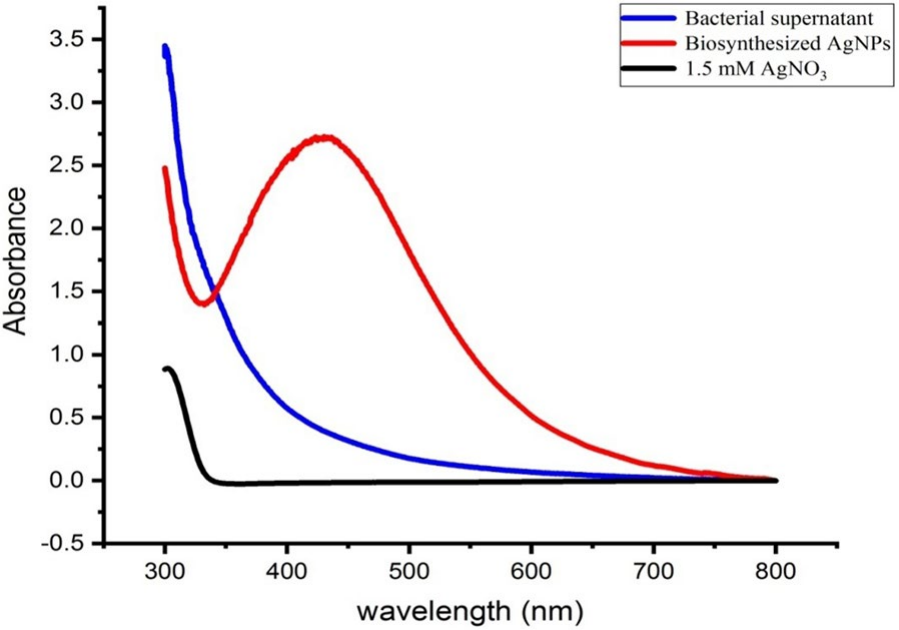
The UV–vis absorption spectra of bio-AgNPs, AgNO_3_, and the supernatant of *Leclercia adecarboxylata* THHM show the SPR peak of bio-AgNPs at 423 nm

The AgNPs were characterized by UV–vis spectroscopy, one of the most widely used techniques for structural characterization of AgNPs. It is generally recognized that UV–vis spectroscopy could be used to verify the synthesis of AgNPs, so the test samples were subjected to UV–vis spectrophotometric analysis. In the UV–vis spectrum of the bio-AgNPs under optimized conditions, a single, strong, and broad peak was observed at 423 nm (Fig. 13), with a slightly red shift than the observed peak of the bio-AgNPs before optimized conditions at 420 nm. Similar results were observed by Akter and Huq [92], who observed a strong peak at around 423 nm of SPR of AgNPs synthesized using *Sphingobium* sp. MAH-11. In addition, similar findings were reported by Abo-State and Partila [93], who biosynthesized AgNPs with strong SPR centred at 423 nm using the supernatant of *Bacillus cereus* MAM-I.11.

### TEM analysis

**Fig. 14:**
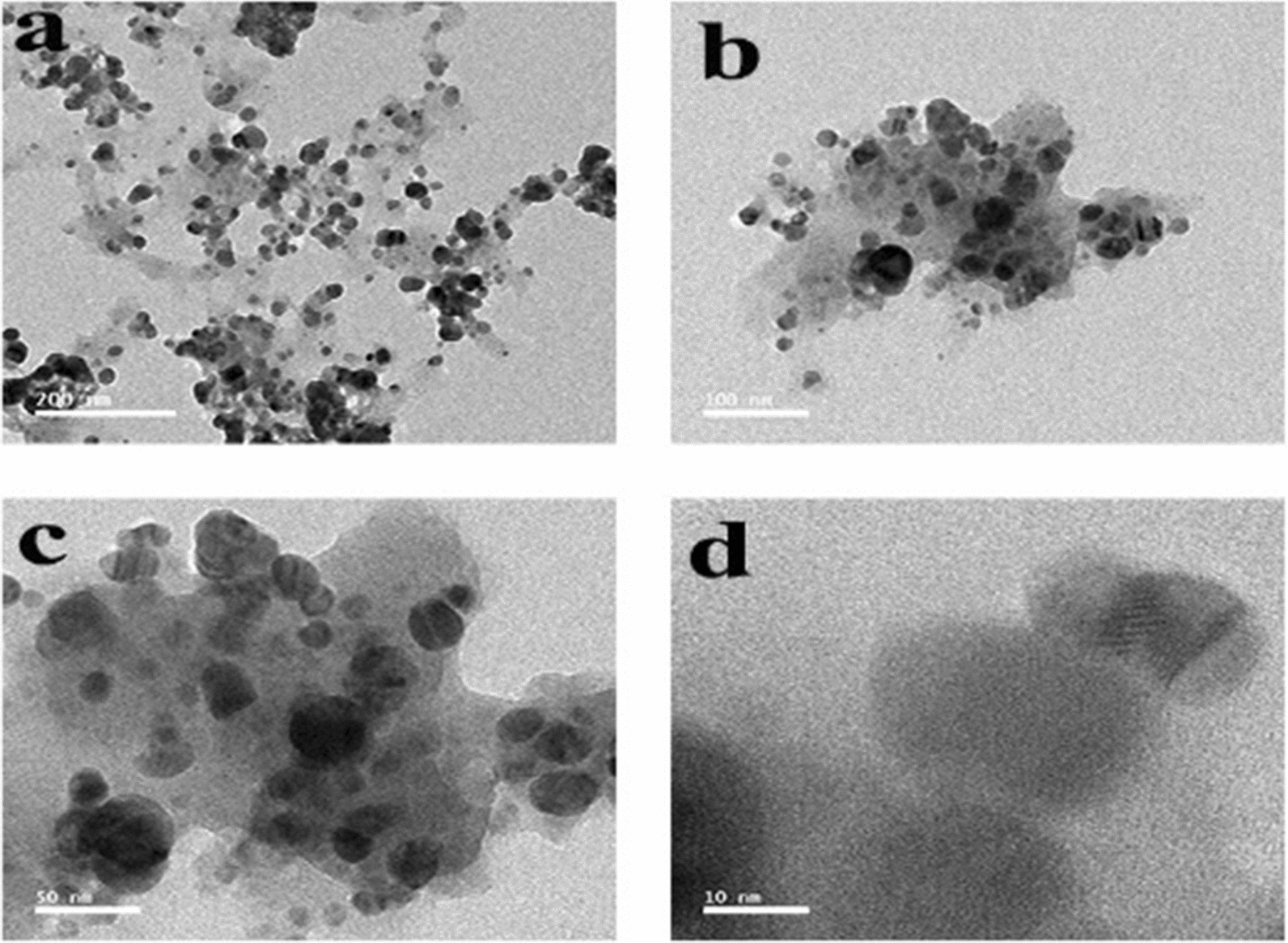
Biosynthesized AgNPs using the supernatant of *Leclercia adecarboxylata* THHM characterized by HRTEM micrographs with different scale bars. **a** 200 nm. **b** 100 nm. **c** 50 nm. **d** 10 nm showing the lattice planes inside the crystal

**Fig. 15:**
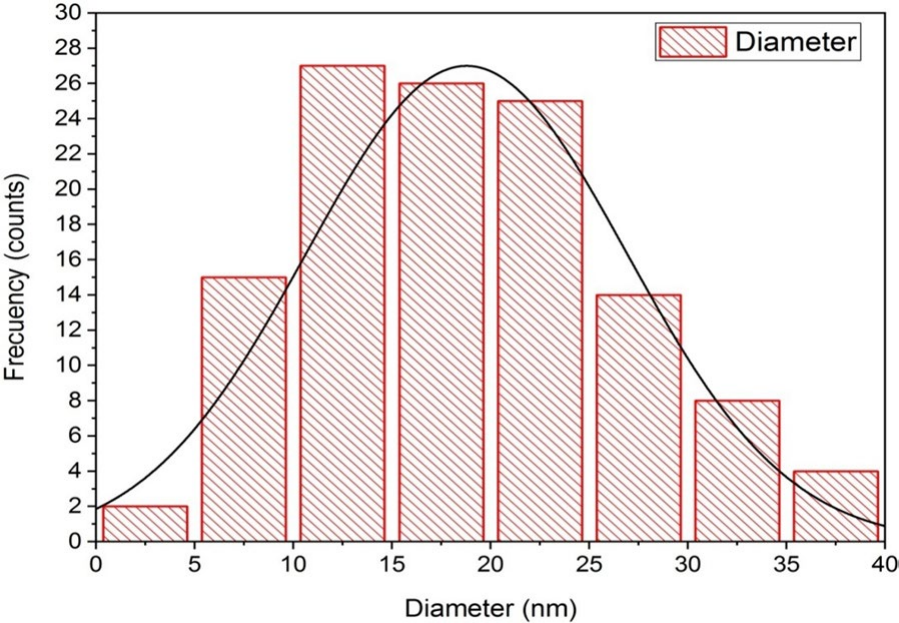
A histogram of the size distribution of the bio-AgNPs

**Fig. 16:**
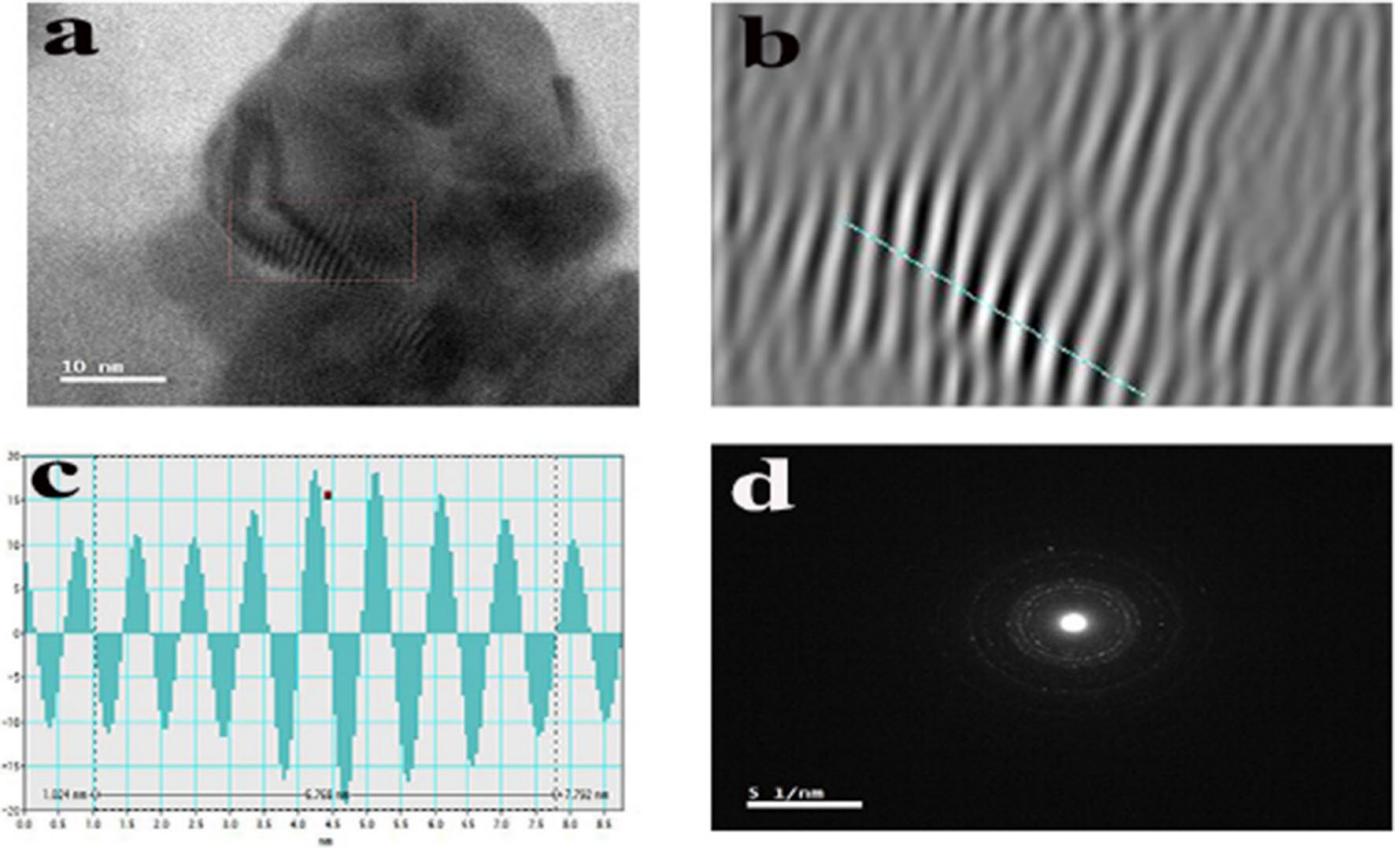
HRTEM micrographs with high resolution of bio-AgNPs and SAED pattern. **a** A crystalline structure of AgNPs with multiple twinning planes. **b** A crystalline structure of AgNPs with a distance between lattice planes inside the crystal. **c** The profile of inverse fast fourier transform with a d-spacing value. **d** SAED pattern with crystal plane spots

TEM is one of the most adapted techniques to study the size and shape of the nanoparticles and provide their distribution [94]. The TEM micrographs of bio-AgNPs confirmed the formation of AgNPs, and these micrographs showed that nanoparticles were generally spherical in shape, well dispersed, and properly separated without any agglomerations (Fig. 14a, b). The TEM images of the particles thus obtained with high magnification power confirmed the spherical morphology of AgNPs (Fig. 14c, d). The particle size of the AgNPs was calculated by ImageJ software with sizes ranging from 3.48 to 39.02 nm with an average particle size of 17.43 nm**.** This was in agreement with TEM observations of Pallavi et al. [3], who found that the AgNPs synthesized using the supernatant of *Streptomyces hirsutus* strain SNPGA-8 obtained by TEM micrograph were spherical in shape with a diameter ranging from 18 to 39 nm. In accordance with our results also, Lotfy et al. [35] reported that AgNPs synthesized using the supernatant of *Aspergillus terreus* showed a spherical shape with a narrow size distribution ranging from 7 to 23 nm. Furthermore, the histogram in Fig. 15 showed a narrow particle size distribution, and the most frequent size of AgNPs was from 10 to 15 nm, measured from more than 100 nanoparticles with a standard deviation of 8.11. Detailed information on the AgNPs microstructure with magnified lattice fringes has been gathered by the fast Fourier transform (FFT) and inverse fast Fourier transform (IFFT) of HRTEM micrographs (Fig. 16a and b). HRTEM micrographs revealed that the AgNPs were crystalline in nature, typically characterized by multiple twinning planes. The obtained results typically resembled the observed results for the crystalline structure of the AgNPs by González-Castillo et al. [95]. The profile of IFFT with a d-spacing value for a specified plane was displayed in Fig. 16c. The analysis of d-spacing values have been carried out by using ImageJ software, which resulted in d_hkl_ values of 0.846 nm for crystal planes on the surface of the AgNPs. The selected area electron diffraction (SAED) pattern of biosynthesized AgNPs (Fig. 16d) contained four spots, each corresponding to specific crystal planes. The SAED pattern from the sample revealed well-defined diffraction spots in the form of rings, which indicate the polycrystalline nature of silver, and with agreement to that found by Murthy et al. [96].

### Fourier transform infrared spectroscopy (FTIR)

**Fig. 17:**
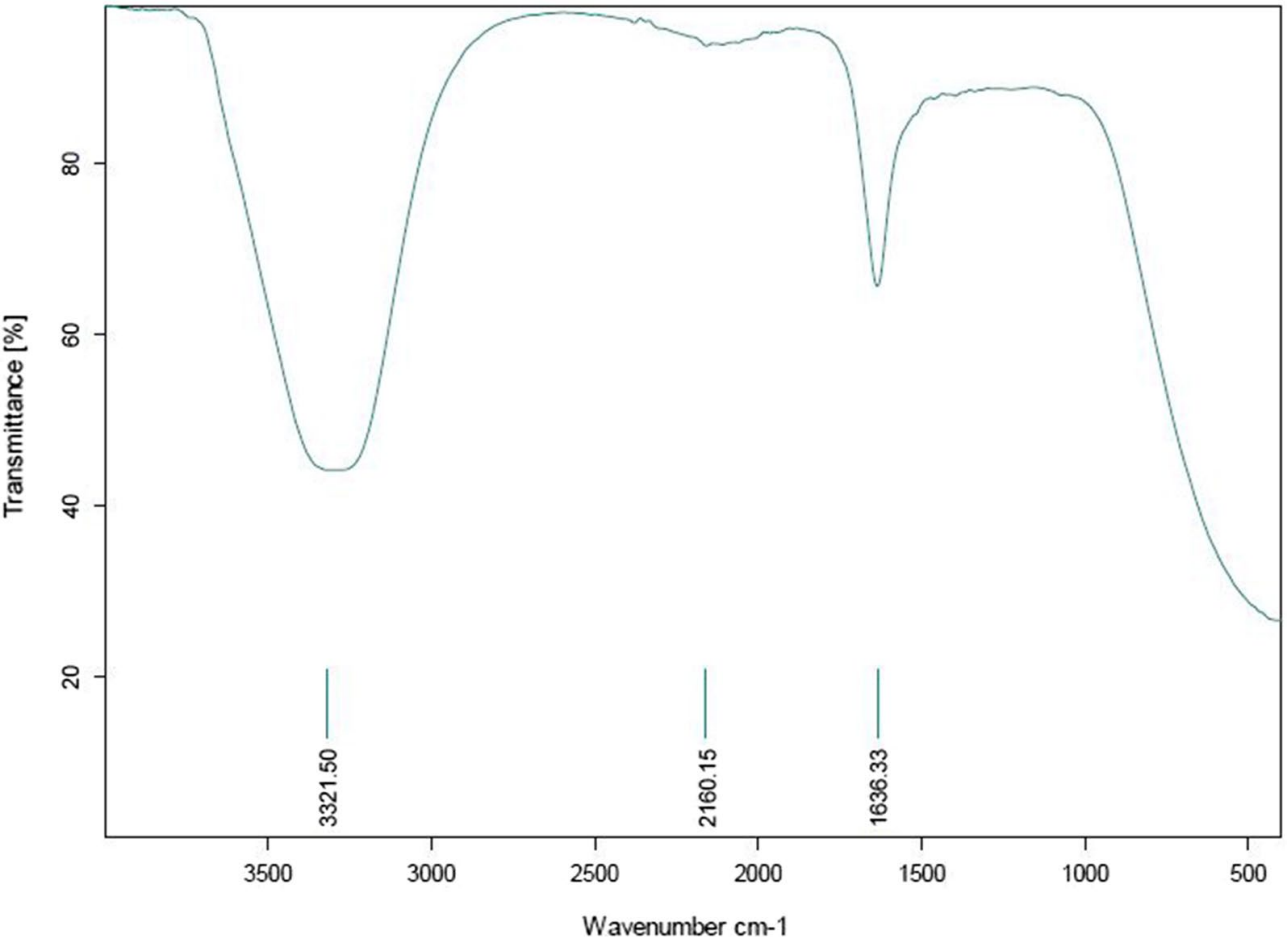
FTIR spectrum of the biosynthesized AgNPs

FTIR analysis was conducted to identify the possible biomolecules that are responsible for capping, reducing, and stabilizing AgNPs [97]. To explore the reduction process of AgNO_3_ by the culture supernatant of *Leclercia adecarboxylata* THHM in the biosynthesized AgNPs, FTIR measurements were carried out to identify possible interactions between silver salts and protein molecules, which could account for the reduction of Ag^+^ ions and stabilization of AgNPs (Fig. 17). In the current study, the FTIR spectra of bio-AgNPs showed three distinct peaks. The position and sharpness of any peak are referred to as group contributions in the synthesis of silver nanoparticles. The sharp peak observed at 3321.50 cm^−1^ was attributed to amine N–H stretching vibrations of peptide linkages and hydroxyl O–H stretching vibrations of carboxylic acid groups, indicating the presence of polyphenols [68, 98]. The broad peak at 2160.15 cm^−1^ was referred to alkynes C≡C stretching and nitrile C≡N groups in aliphatic/aromatic compounds [99]. The sharp peak at 1636.33 cm^−1^ has been attributed to the N–H primary amine group; however, it could also be due to carbonyl C = O stretch in polyphenols [98] or alkene C = C groups from aromatic compounds [100]. The overall finding confirms the presence of proteins in the bio-AgNPs samples. According to earlier reports [65], proteins can bind to nanoparticles through their free amine groups or cysteine residues. Therefore, stabilization of AgNPs by proteins is a clear possibility.

Our findings were in agreement with those obtained by Sarsar et al. [67], who reported that the FTIR spectrum of biosynthesized AgNPs using the filtrate extract of novel fungal strain *Penicillium atramentosum* KM showed three distinct peaks, 3360.72, 1643.82, and 462.86 cm^−1^, which are attributed to the stretching vibrations of primary amines, carbonyl stretch vibrations in the amide linkages of proteins, and the fingerprint, respectively. Mobaraki et al. [101] reported that the obtained biosynthesis AgNPs using green tea have similar characteristic peaks at 3441 and 1635 cm^−1^ for stretching vibrations of the O–H and N–H primary amine groups, respectively.

### The antimicrobial activity of the synthesized AgNPs

**Fig. 18:**
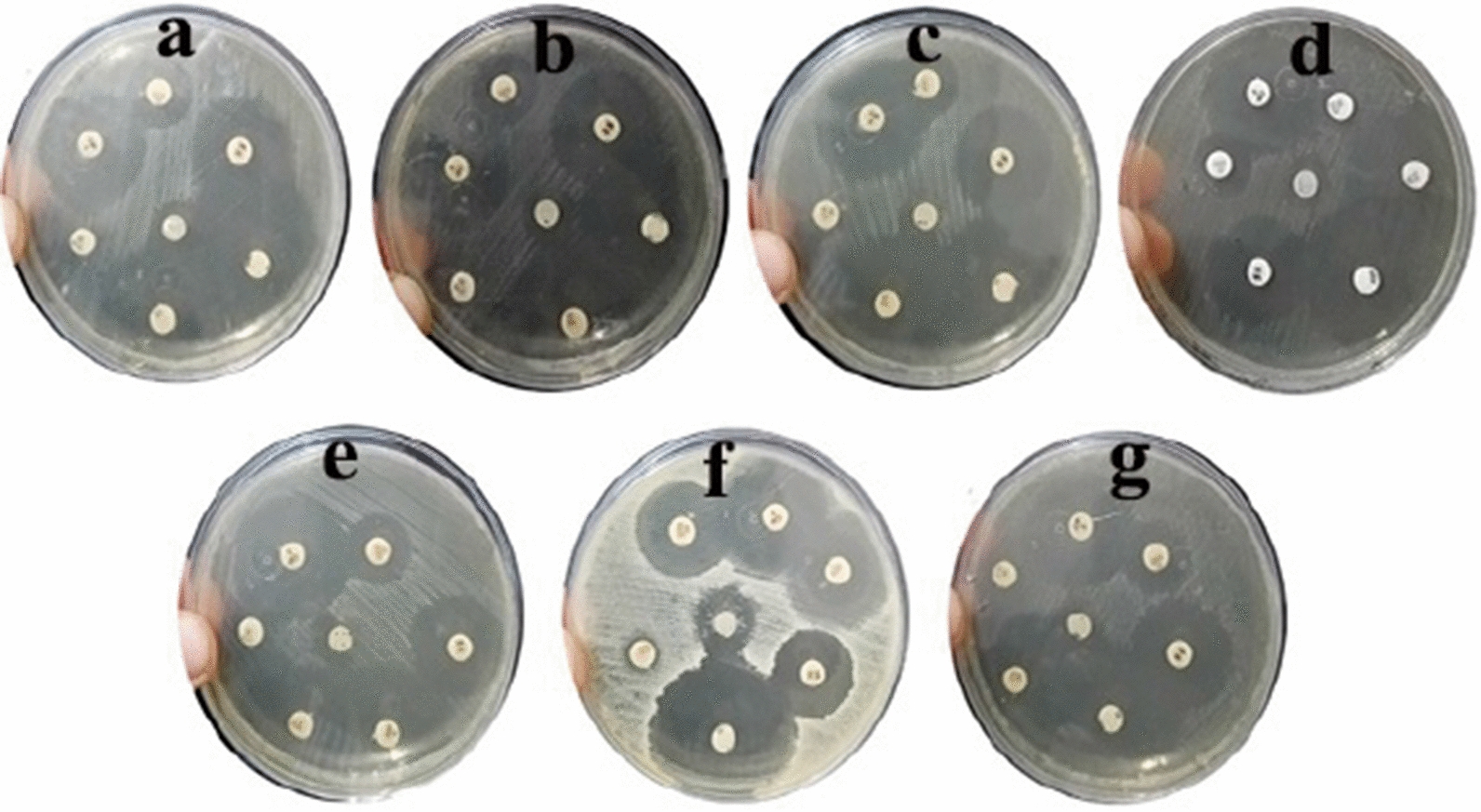
Antimicrobial activity of bio-AgNPs (center disc) at a concentration of 1000 µg/mL against human pathogenic microbes. **a**: *Staphylococcus aureus* ATCC6538, **b**: *Bacillus cereus* ATCC6633, **c**: *Escherichia coli* NCTC10418*,* **d**: *Vibrio cholera* ATCC700, **e**: *Pseudomonas aeruginosa* ATCC9027, **f**: *Klebsiella pneumoniae* ATCC13883, and **g**: *Candida albicans* ATCC700

The bio-AgNPs were examined for their antimicrobial activity compared with six different antibiotics belonging to six different antibiotic classes, which were used as positive controls. The antimicrobial activity of the AgNPs and the antibiotics was investigated against seven clinically isolated pathogens using the disc diffusion method by an inhibitory zone [102]. As indicated in Fig. 18 and Table 3, the effect of the bio-AgNPs on the seven tested microbes was slightly varied. The most affected bacterial pathogen was *Vibrio cholera* ATCC700 strain, which was highly affected compared to *Pseudomonas aeruginosa* ATCC9027 strain, which was the least affected one. The bio-AgNPs were able to form clear zones with 16 mm and 10 mm diameters for *Vibrio cholera* ATCC700 and *Pseudomonas aeruginosa* ATCC9027 strains, respectively. However, it showed the same antimicrobial pattern towards *Staphylococcus aureus* ATCC6538 and *Klebsiella pneumoniae* ATCC13883 group or *Bacillus cereus* ATCC6633, *Escherichia coli* NCTC10418, and *Candida albicans* ATCC700 group. Their effect on *Staphylococcus aureus* ATCC6538 and *Klebsiella pneumoniae* ATCC13883 was somehow higher than that recorded for *Bacillus cereus* ATCC6633, *Escherichia coli* NCTC10418, and *Candida albicans* ATCC700. It almost showed a 14 mm clear zone against the first group and a 12 mm clear zone against the second. It is worth mentioning that the bio-AgNPs’ pattern of activity against the tested pathogens was as follows: *Vibrio cholera* ATCC700 > *Staphylococcus aureus* ATCC6538 = *Klebsiella pneumoniae* ATCC13883 > *Bacillus cereus* ATCC6633 = *Escherichia coli* NCTC10418 = *Candida albicans* ATCC700 > *Pseudomonas aeruginosa* ATCC9027. A wide variation was found in the activities of the various types of antibiotics against the seven microbial pathogens, which is interpreted in terms of the diameter of zones of inhibition. The results showed that ampicillin (10 µg) had the highest overall antimicrobial activity against the seven microbial pathogens, followed by tetracycline (30 µg), ciprofloxacin (5 µg), vancomycin (30 µg) gentamicin (10 µg), and ceftriaxone (30 µg) as shown in Fig. 18 and Table 3. The results were comparable and followed the previous studies based on the antimicrobial activity of AgNPs [67, 103]. Our findings of the antimicrobial activity are corroborated by the earlier report of Ma et al. [61] wherein they investigated the antimicrobial activity of AgNPs against *Escherichia coli* ATCC-8739, *Pseudomonas aeruginosa* ATCC-15442, *Staphylococcus aureus* ATCC-6538, *Bacillus subtilis* ATCC-6633, and *Candida albicans* ATCC-10231. In agreement with our results, Bawskar et al. [104] reported that antimicrobial assay against *Escherichia coli* and *Staphylococcus aureus* has proven that the biosynthesized AgNPs using the culture filtrate of *Fusarium oxysporum* and the leaf-extract of *Azadirachta indica* have a potent antibacterial activity. Similarly, Ghetas et al. [105] reported that the biologically produced AgNPs using leaves extract of *Origanum vulgare* showed antimicrobial activity against *Streptococcus agalactiae*, *Aeromonas hydrophila*, and *Vibrio alginolyticus*. Several mechanisms have been reported for the antimicrobial activity of AgNPs, but the exact mechanism has not been established yet [77].

**Table 3:** Measurements of clear zones obtained by bio-AgNPs and different commercially antibiotics against seven human pathogenic microbes

Microbial strain	Clear zone (mm)						
	bio-AgNPs	Antibiotic					
		GEN	AMP	CTR	VA	CIP	TE
*Staphylococcus aureus* ATCC6538	14	16	36	0	26	26	34
*Bacillus cereus* ATCC6633	12	16	36	0	26	30	36
*Escherichia coli* NCTC10418	12	16	36	0	26	30	34
*Vibrio cholera* ATCC700	16	16	34	15	24	30	34
*Pseudomonas aeruginosa* ATCC9027	10	18	34	0	26	30	33
*Klebsiella pneumoniae* ATCC13883	14	16	38	22	22	32	10
*Candida albicans* ATCC 700	12	16	36	12	24	26	35

Where *bio-AgNPs* biosynthesized AgNPs, *GEN* gentamicin (10 µg), *AMP* ampicillin (10 µg), *CTR* ceftriaxone (30 µg), *VA* vancomycin (30 µg), *CIP* ciprofloxacin (5 µg), and *TE* tetracycline (30 µg)

### The minimum inhibitory concentrations (MICs) of bio-AgNPs

**Fig. 19:**
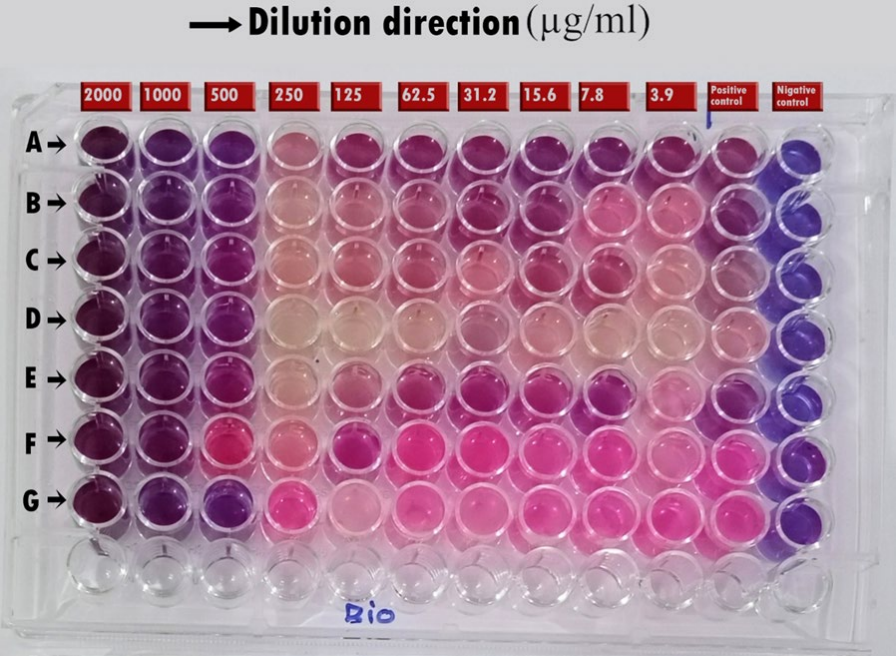
Determination of MIC values using resazurin microtiter plates of bio-AgNPs against the tested human pathogenic microbes. **A**: *Staphylococcus aureus* ATCC6538, **B**: *Bacillus cereus* ATCC6633, **C**: *Escherichia coli* NCTC10418*,* **D**: *Vibrio cholera* ATCC700, **E**: *Pseudomonas aeruginosa* ATCC9027, **F**: *Klebsiella pneumoniae* ATCC13883, and **G**: *Candida albicans* ATCC700. The numbers above the plate represent the concentration of AgNPs (µg/mL) applied to each well of the column. Positive control: without the addition of AgNPs, Negative control: only LB broth

The antimicrobial activity and potency of AgNPs have been quantitatively assessed by determining the MIC values [106]. The MIC is defined as the minimum concentration of the antibiotic substance required to inhibit the growth of microbial pathogens compared to the control [3, 106, 107]. Freshly grown cultures of the tested microbial pathogens diluted in LB broth were exposed separately to bio-AgNPs at concentrations ranging from 2000 μg/mL to 3.9 μg/mL to determine the MICs of the tested pathogens using a resazurin-based microtiter dilution assay. The used procedure mainly depends on the microbial metabolism that is responsible for converting the blue color of resazurin to pink. Resazurin, a redox-sensitive dye, was used as an indicator to determine cell growth and to verify cell viability [106, 108]**.** The non-fluorescent blue resazurin is reduced to fluorescent red resorufin by oxidoreductases found in viable cells [108]. This fluorescence and the visible change in color indicate that the cells are viable. Dead cells do not reduce resazurin and are indicative of cell death [109]. As shown in Fig. 19 and Table 4, it was found that all the microbial pathogens have the same MIC value of 500 µg/mL with bio-AgNPs except for *Klebsiella pneumoniae* ATCC13883, which has a higher MIC value of 1000 µg/mL. Similar and comparative observations have been documented in previous studies; as the biosynthesized AgNPs have demonstrated notable MIC values against pathogens by effectively inhibiting the bacterial growth [3, 110]. It has been reported that biomolecules acting as stabilizing agents give better antibacterial activity for biosynthesized AgNPs [5, 111]. The equivalent and comparing observations were recorded in the earlier study of Pallavi et al. [3], who reported that the biosynthesized AgNPs manifested notable MIC values against many microbial pathogens. Our results are consistent with those previously reported by Bhat et al. [110] who mentioned that the MIC of biosynthesized AgNPs was determined for four bacterial pathogens including *Staphylococcus aureus* MTCC6908, *Bacillus subtilis* MTCC2393, *Pseudomonas aeruginosa* MTCC424, and *Escherichia coli* MTCC40. The lowest MIC value was recorded for *Bacillus subtilis* MTCC2393 at 6 µg/mL, while the highest MIC value was recorded for *Pseudomonas aeruginosa* MTCC424 at 9 µg/mL. The mechanism behind the antibacterial activity of AgNPs may be dependent on the capping and the concentration of the AgNPs and their mode of entry into the bacterial cell [104].

**Table 4:** The MIC of bio-AgNPs responsible for ceasing the growth of the tested human pathogenic microbes

Microbial strain	MIC (µg/mL)
	bio-AgNPs
*Staphylococcus aureus* ATCC6538	500
*Bacillus cereus* ATCC6633	500
*Escherichia coli* NCTC10418	500
*Vibrio cholera* ATCC700	500
*Pseudomonas aeruginosa* ATCC9027	500
*Klebsiella pneumoniae* ATCC13883	1000
*Candida albicans* ATCC 700	500

### Suggested antibacterial mechanisms of biosynthesized silver nanoparticles

The chemical or biological production of AgNPs is mainly depending on the reduction of Ag^+^ ions into Ag^o^ particles. And hence, the antibacterial activity of AgNPs is basically assigned to its Ag^o^ basis [112]. The formed AgNPs are known for their small-scale dimensions that help for their attachment to the bacterial cell walls followed by penetration inside the bacterial cells [113]. The silver particles are subsequently bind with high affinity to the bacterial proteins, and resulting in the damaging of the DNA followed by the inhibition of the bacterial proliferation which eventually leads to their death [114]. Furthermore, the cell wall and cell membrane are the first barriers that help bacteria to resist the external environments. As known in Gram-negative bacteria, the cell wall lacks a thick peptidoglycan layer, which in turn increases the bactericidal action and causes the generation of more reactive oxygen species (ROS), especially when exposed to nanoparticles that have a higher specific area to volume ratio [115, 116]. The formed superoxide and hydroxide ions have a negative charge that prevents them from permeating into cells, but they remain on the bacterial cell walls and disrupt their completeness, which leads to the damage of the cell wall with subsequent discharging of intracellular contents, causing cell death [117]. In addition, hydrogen peroxide is able to permeate into the cell and damage its respiratory enzymes, causing cell death as well [118]. However, it has been reported that the green synthesis of nanoparticles has resulted in the capping of the produced nanoparticles, which helps to prevent their toxicity and side effects when applied in the field of animal tissue culture [101].

## Conclusion

The biosynthesis provides an eco-friendly, cost-effective and efficient approach for the production of AgNPs, which could act as excellent antimicrobial agents against pathogenic microbes. The present study has isolated a strain of *Leclercia adecarboxylata* THHM (accession number OK605882) from soil, which showed the capability of reducing Ag ions into AgNPs extracellularly with the formation of a characteristic peak at 423 nm. The biosynthesized AgNPs were characterized by UV–visible spectroscopy, TEM, and FTIR. The AgNPs exhibited a uniform morphology of a spherical nature with sizes ranging from 3.48 to 39.02 nm with an average particle size of 17.43 nm**.** FTIR analysis revealed that different functional groups present in the supernatant of *Leclercia adecarboxylata* THHM caused the reduction of silver ions and helped in the formation of nanoparticles in the biosynthesis procedure. The PBD was applied to optimize the biosynthesis of AgNPs and showed that the variables illumination, AgNO_3_ concentration, bacterial supernatant concentration, and time have a significant positive effect on the production of nanoparticles, while the variable pH showed a negative effect. The biosynthesized AgNPs were found to be effective against important clinical pathogens, including *Staphylococcus aureus* ATCC6538, *Bacillus cereus* ATCC6633, *Escherichia coli* NCTC10418*, Vibrio cholera* ATCC700, *Pseudomonas aeruginosa* ATCC9027, *Klebsiella pneumoniae* ATCC13883, and *Candida albicans* ATCC700 with MIC values of 500 µg/mL for all microbial pathogens and 1000 µg/mL for *Klebsiella pneumoniae* ATCC13883. This report presents data for the first time on the ability of the supernatant of *Leclercia adecarboxylata* THHM under optimum conditions to produce AgNPs of small size that having an acceptable antimicrobial activity against important clinical pathogens.

## Materials and methods

### Isolation of bacteria

Bacterial isolates were extracted from twenty soil samples, which were collected from different locations in Kafr El-Sheikh city. The soil samples were collected from the soil surface (0–5 cm) and at a depth of approximately 20 cm. All samples were immediately transferred into sterilized polyethylene bags using sterilized spatulas and were subsequently stored at 4 ºC until examination. Each soil sample was passed through a sieve (1.7 mm mesh) to remove large pieces. The samples were diluted serially in sterile 0.8% NaCl. About 50 µL of each dilution was spread over the surface of nutrient agar (Peptone 5.0 g, Beef extract 3.0 g, Sodium chloride 8.0 g, Agar 16.0 g, and 1000 mL distilled water, pH 7.3 ± 0.2) plates using a sterile glass spreader [119]. After complete dryness, the plates were incubated at 30 °C for 24 h. Single colonies that represent every unique colony type were chosen from each plate based on colonial morphology. The isolated colonies were obtained in the form of pure cultures and were latterly screened to explore their potential to form AgNPs. The bacterial isolates were either kept separately on nutrient agar at 4 °C and recultured every 4 weeks and/or were inoculated into sterile 60% glycerol and kept for longer periods at −20 °C.

### Screening of the most potent AgNPs-producing bacteria

The obtained bacterial isolates were screened for their ability to produce AgNPs as follows:

### Preparation of cell free extract

One milliliter of overnight culture of each bacterial isolate was inoculated into 250 mL Erlenmeyer flask containing 100 mL of sterile LB broth (Tryptone 10.0 g, Yeast extract 5.0 g, and 1000 mL distilled water, pH 7.5 ± 0.2) free from NaCl. Inoculated flasks were incubated in a rotating shaker set at 200 rpm for 72 h at 30 ^ο^C. After incubation, each culture was centrifuged at 12,000 rpm for 10 min, and its supernatant was used for further experiments [8, 49].

### Biosynthesis of silver nanoparticles (AgNPs)

The supernatant from each isolate was investigated for extracellular synthesis of AgNPs. A total volume of 50 mL of each supernatant was mixed separately with filter-sterilized AgNO_3_ aqueous solution at a 1.0 mM final concentration. All the reaction mixtures were incubated on a rotating shaker at 200 rpm at 30 ^ο^C for 72 h in the dark. Control experiments were performed with un-inoculated media and silver nitrate solution to check the role of bacteria in the synthesis of nanoparticles. Visual observation was conducted periodically to check for the synthesis of AgNPs by color change [32]. The reduction of Ag^+^ ions was monitored by a visual color change to yellowish brown and its intensity was spectrophotometrically determined by using a UV–vis spectrophotometer (Double Beam Spectrophotometer 6800 JENWAY) in the range of 200–800 nm [49].

### Molecular identification of the targeted isolate

The selected bacterial isolate that showed a potent ability to biosynthesize AgNPs was subjected to DNA extraction, PCR amplification, and sequencing of its 16S rRNA gene. The genomic DNA of the bacterial isolate was extracted using a DNA extraction kit (Qiagen, USA) according to the manual instructions. The DNA extracted by the previous step was used as a template for the PCR amplification of the 16S rRNA gene using universal primers. The sequence of the forward primer was 5'-ACTCCTACGGGAGGCAGCAG-3' and the sequence of the reverse primer was 5'-CCGTCAATTCATTG-3'. The PCR was carried out in a total volume of 50 µL containing 10 ng of genomic DNA, 30 pmol of each primer, 2.5 units of *Taq* DNA polymerase, 10 mM of each dNTPs and 1X PCR buffer as components. The PCR was carried out starting with a denaturation step at 94 °C for 5 min, followed by 30 cycles at 94 °C for 1 min, 55 °C for 1 min, and 72 °C for 2 min, followed by a final extension step at 72 °C for 10 min. After program completion, a fraction of the PCR mixture was examined using agarose gel electrophoresis [120], and the remnant was purified to remove excess primers and nucleotides using QIAquick PCR purification reagent (Qiagen, USA) and was submitted for sequencing (Sigma, Germany). The obtained sequence was then compared with a database library by using analysis software. The Blast program was used to assess the DNA similarities, and multiple sequence alignment and molecular phylogeny were performed using the MEGA 11.0 program [121].

### Optimization of the biosynthesis of AgNPs

Different reaction parameters may have a variable effect on the reduction process of a metal ion and probably alter the shape and size of the final product [1]. In reference to the above-mentioned statement, the factorial design of experiments, the one variable-at-time (OVAT) method, was used. The investigational factors were changed one at a time, with the left-over factors remaining constant. The different parameters, such as contact time, silver nitrate concentration, pH, reaction temperature, and supernatant percentage, were optimized to obtain the maximum, rapid, and stable biosynthesized AgNPs. All of the reaction mixtures were analyzed by a UV–vis spectrophotometer at 420 nm.

### Contact time

The reaction time of the biosynthesis of AgNPs was optimized as previously described [49]. The reaction time was monitored at different time intervals from 0.0 to 72.0 h to determine the time needed for maximum production of AgNPs. About 250 mL of the selected bacterial isolate supernatant at pH 7.0 was mixed with filter-sterilized AgNO_3_ aqueous solution at 1.0 mM final concentration. The reaction mixture was incubated at 30 ^ο^C in the dark. The absorbance of the resulting solution was measured spectrophotometrically by sampling about two mL of the reaction mixture at the end of each time interval.

### Concentration of silver nitrate

The effect of different AgNO_3_ concentrations on the biosynthesis of AgNPs was optimized as previously described by Gurunathan et al. [65], with a few modifications. At pH 7.0, the supernatant of the selected bacterial isolate was treated with AgNO_3_ at final concentrations ranging from 1.0 to 6.0 mM to determine the optimum concentration that yields the maximum and stable production of AgNPs. After incubation for 48.0 h at 30 ^ο^C, the absorbance of the resulting solutions was measured spectrophotometrically.

### pH

The pH of the reaction mixture used in the biosynthesis of AgNPs was optimized using different pH values, where the reaction pH was adjusted at 4.0, 5.0, 6.0, 7.0, 8.0, 9.0, and 10.0. The supernatant of the selected bacterial isolate was adjusted at different pH values and treated separately with AgNO_3_ at a final concentration of 1.0 mM. The pH of the reaction mixtures was maintained with the help of 0.1 N HCl and 0.1 N NaOH solutions. After incubation of 48.0 h at 30 ^ο^C, the absorbance of the resulting solutions was measured spectrophotometrically [60, 122].

### Temperature

The effect of different temperatures on the rate of biosynthesis of AgNPs was studied. At pH 7.0, with AgNO_3_ at 1.0 mM final concentration, the temperatures of the reaction mixtures of the supernatant of the selected bacterial isolate were investigated by incubating at (30, 37, 40, and 45 ºC) for 48.0 h. The absorbance of the resulting solutions was measured spectrophotometrically [67, 123].

### Bacterial supernatant concentration

The effect of different supernatant concentrations of the bacterial isolate on the biosynthesis of AgNPs were investigated as described by Ma et al. [61] with some modifications. The supernatant of the bacterial isolate was dispensed into six Erlenmeyer conical flasks, and their concentrations were adjusted as 10, 20, 40, 60, 80, and 100% (v/v) using sterilized distilled water. At pH 7.0, all Erlenmeyer conical flasks were treated with a filter sterilized AgNO_3_ aqueous solution at a final concentration of 1.0 mM. After incubation at 40 ^ο^C for 48.0 h, the absorbance of the resulting solutions was measured spectrophotometrically.

### Optimization of the biosynthesis of AgNPs using Plackett–Burman design (PBD)

Plackett–Burman fractional factorial design [124, 125] was used to investigate various factors that affect the biosynthesis of AgNPs by the supernatant of the selected bacterial isolate. The five independent variables chosen for the current study were AgNO_3_ concentration, supernatant concentration, incubation time, pH, and illumination. They were designated as A, B, C, D, and E, respectively, and for each variable, high (+ 1) and low (−1) levels were tested (Table 5). The upper and lower limits of the analysis were decided after a series of earlier optimization experiments. The five independent variables were organized according to the Plackett–Burman design matrix in eight trials. Each variable was examined in four trials at high level and in four trials at low level. All trials were performed in duplicate to minimize experimental errors. The biosynthesis of AgNPs was determined by measuring the absorbance of the resultant solutions spectrophotometrically at 420 nm, and the average absorbance of the biosynthesized AgNPs was considered as a response. The main effect of each variable was determined using the following equation:

$${\mathbf{E}}_{{{\mathbf{xi}}}} = \, \left( {\sum {\mathbf{M}}_{{{\mathbf{i}} + }} - \, \sum {\mathbf{M}}_{{{\mathbf{i}} - }} } \right) \, / \, {\mathbf{N}}$$

**Table 5:** Experimental independent variables at two levels used for the biosynthesis of AgNPs by the supernatant of the selected bacterial isolate using Plackett–Burman design

Variable	Symbol	level	
		Low (−1)	High (+ 1)
AgNO_3_ conc.	A	0.5 mM	1.5 mM
Bacterial supernatant conc.	B	10%	30%
incubation Time	C	24.0 h	72.0 h
pH	D	7.0	9.0
Illumination	E	Dark	Light

where E_xi_ was the variable's main effect, M_i+_ and M_i−_ were the absorbance of the biosynthesis of AgNPs in trails where the independent variable (xi) was present in high and low concentrations, respectively, and N is the number of trials divided by 2. A main effect figure with a positive sign indicates that the high concentration of this variable is near to the optimum and a negative sign indicates that the low concentration of this variable is near to the optimum. Based on the biosynthesized AgNPs, the factorial experiment was analyzed using regression analysis and ANOVA. From the regression analysis, the variables that were significant (*P* < 0.05) were considered to have a greater impact on the biosynthesized AgNPs [126]. Using Microsoft Excel, the statistical *t*-value for equal unpaired samples, *P*-value, and confidence level were calculated for the determination of the significance of the variables [35, 125].

### Biosynthesis of AgNPs under optimized conditions

After optimization of various parameters for the biosynthesis of AgNPs, the bio-reduction of AgNO_3_ was carried out using the supernatant of the selected bacterial isolate under optimum conditions. The resulting reaction mixture was centrifuged at 10,000 rpm for 30 min in order to obtain the pellets of AgNPs. The produced pellets were re-suspended in distilled water and further centrifuged. The pellets were washed thoroughly at least three times with distilled water to remove any biological contaminants present. The AgNPs were subsequently dried and stored for further use [1].

### Characterization of the prepared AgNPs

Preliminary characterization of the biosynthesized AgNPs under optimized conditions (bio-AgNPs) was performed through visual observation for changes in color. The appearance of a yellow to brown color of the reaction mixtures indicates the formation of AgNPs. The synthesized AgNPs were further characterized using ultraviolet–visible (UV–vis) spectroscopy, transmission electron microscopy (TEM), and Fourier transform infrared (FTIR) spectroscopy.

### Ultraviolet–visible (UV–vis) spectral analysis

The formation of AgNPs by reduction of AgNO_3_ in colloidal solution was monitored using UV–vis spectral analysis. Biosynthesized AgNPs absorption peaks were observed in the UV–vis spectrophotometer (Double Beam Spectrophotometer 6800 JENWAY, Japan) by scanning the absorbance spectra in the 200–800 nm range of wavelength at a resolution of 0.5 nm [127].

### Transmission electron microscopy (TEM) analysis

The TEM technique was employed to visualize the morphology and size of the biosynthesized AgNPs. The 200 kV high-resolution transmission electron microscope (HRTEM) (JEOL-2010) was used. TEM grids were prepared by placing a drop of the particle solution on a carbon-coated copper grid and drying under a lamp. TEM images of AgNPs were obtained at an accelerating voltage of 120 kV [65]. The average diameter and size distribution of nanoparticles were obtained from TEM micrographs by using image analysis software (ImageJ). Values and size distributions were calculated with the mean and standard deviation.

### Fourier transform infrared (FTIR) spectroscopy

FTIR spectra were used to investigate the functional groups on the surface of the biosynthesized AgNPs under optimized conditions. In FTIR analysis, the biotransformed AgNPs sample was prepared by uniform dispersion in a dry KBr powder and compression to form a disc. A disc of 50 mg of KBr was prepared with a mixture of 2% finely dried sample. The composition of these powders and the surface functional groups were examined under a Spectrum One Spectrophotometer (Perkin Elmer, USA). An infrared spectrum was recorded in the region of 400–4000 cm^−1^ with a resolution of 4 cm^−1^ [128].

### Determination of the antimicrobial activity of the biosynthesized AgNPs compared with commercially available antibiotics

The agar disc diffusion method was employed to determine the antimicrobial activities of the biosynthesized AgNPs against clinically isolated pathogens [129]. In bacterial pathogens, Gram-positive bacteria such as *Staphylococcus aureus* ATCC6538 and *Bacillus cereus* ATCC6633 and Gram-negative bacteria such as *Escherichia coli* NCTC10418*, Vibrio cholera* ATCC700, *Pseudomonas aeruginosa* ATCC9027, and *Klebsiella pneumoniae* ATCC13883 were used. In addition to the fungal pathogen *Candida albicans* ATCC700 as a representative unicellular fungus. Clinical microbial isolates were kindly provided by the Environmental Biotechnology Department, Genetic Engineering and Biotechnology Research Institute (GEBRI), City of Scientific Research and Technological Applications (SRTA-City), New Borg El-Arab City, 21934, Alexandria, Egypt. Initially, LB broth (Tryptone 10.0 g, Yeast extract 5.0 g, Sodium chloride 10.0 g, and 1000 mL distilled water, pH 7.5 ± 0.2) and LB agar (Tryptone 10.0 g, Yeast extract 5.0 g, Sodium chloride 10.0 g, Agar 15.0 g, and 1000 mL distilled water, pH 7.5 ± 0.2) were prepared and sterilized at 121 °C and 15 psi for 20 min. Pure colonies of the above mentioned microbial strains were cultured in 5 mL of LB broth and were incubated for 18 h with shaking at 200 rpm at 30 °C. After incubation, a 0.5 McFarland standard was prepared for each strain, and 100 μL of each culture was separately plated uniformly on the LB agar plates using sterile cotton swabs. Sterilized circular paper discs with a diameter of 6 mm were placed in the center of Petri plates in contact with the culture. Then, about 25 µL of 1000 μg/mL of biosynthesized AgNPs were carefully pipetted onto the discs. Commercially available antibiotic discs belonging to different antibiotic classes, including gentamicin (10 µg), ampicillin (10 µg), ceftriaxone (30 µg), vancomycin (30 µg), ciprofloxacin (5 µg), and tetracycline (30 µg) were placed on the plates all around the previous discs. The antimicrobial activities of the antibiotic discs were used as a positive control to be compared with the activity of AgNPs. The plates were then kept for 1 h at 4 °C before being incubated for 24 h at 30 °C. The diameter of the inhibition zones was checked, measured in millimeters, and photographed. The experiments were carried out in duplicates.

### Determination of the minimum inhibitory concentrations (MICs) of the synthesized AgNPs

The MIC is the lowest concentration of an antimicrobial agent required to inhibit the visible growth of an organism. The ability of different concentrations of the biosynthesized AgNPs to inhibit the growth of the tested microbial pathogens was examined. The microbial pathogens utilized for the test were *Staphylococcus aureus* ATCC6538, *Bacillus cereus* ATCC6633, *Escherichia coli* NCTC10418*, Vibrio cholera* ATCC700, *Pseudomonas aeruginosa* ATCC9027, *Klebsiella pneumoniae* ATCC13883, and *Candida albicans* ATCC700. The MIC for each strain was determined by using the broth microdilution method [107]. This was accomplished by tracing the color change in the resazurin indicator from a blue/non-fluorescent state to a pink/highly fluorescent state that indicates the microbial metabolism, and hence, growth. Initially, 100 µL of the biosynthesized AgNPs from 2000 µg/mL of stock solutions were instilled into the first column of a 96-well microtiter plate, while 50 µL of the LB broth were added to the rest of the columns. A series dilution of two folds then proceeded horizontally, transferring and mixing 50 µL of each well in the first column to the second well, continuously up to the tenth columns. After that, 50 µL of 0.5 McFarland standard of each microbial strain was added to all wells within the same row until the tenth column, followed by the addition of 100 µL LB broth. Column 11 (medium with bacterial inoculum) wells were composed of 100 µL of 0.5 McFarland standard of each microbial strain, plus 100 µL of LB broth, and column 12 (only medium) wells were filled with 200 µL of LB broth, serving as positive and negative controls, respectively. The highest concentration of AgNPs was contained in column 1, whereas the lowest concentration was contained in column 10. The plate was incubated at 30 °C for 24 h, followed by the addition of 40 µL of resazurin (0.015%) to each well. All wells were checked for color changes after 2 h of incubation at 30 °C. The lowest concentration of AgNPs at which the resazurin blue color remained unchanged was recorded as the MIC [109].

### Statistical analysis

The obtained data were statistically analyzed using OriginPro 2018 software (Origen Lab Corporation, Northampton, Massachusetts, USA) and the Microsoft Excel^®^ Program. All values in the experiments were expressed as the mean ± standard deviation (SD) and were analyzed with one-way Analysis of Variance (ANOVA). The significant level was set at *p* < 0.05.

## Data Availability

All datasets contained in this study are listed in the manuscript.

## Abbreviations

AgNO_3_: Silver nitrate
AgNPs: Silver nanoparticles
ANOVA: Analysis of variance
bio-AgNPs: Biosynthesized AgNPs
FFT: Fast Fourier transform
FTIR: Fourier transform infrared spectroscopy
HRTEM: High-resolution transmission electron microscopy
IFFT: Inverse fast Fourier transform
LB: Luria–Bertani
MIC: Minimum inhibitory concentration
OVAT: One variable at a time
PBD: Plackett–Burman experimental design
PCR: Polymerase chain reaction
SAED: Selected area electron diffraction
SPR: Surface plasmon resonance
TEM: Transmission electron microscopy
UV–Vis: Ultraviolet–visible spectroscopy

## References

[1] Verma A, Tyagi S, Verma A, Singh J, Joshi P (2017). Optimization of different reaction conditions for the bio-inspired synthesis of silver nanoparticles using aqueous extract of *Solanum nigrum* leaves. J Nanomater Mol Nanotechnol.

[2] Ahmad S, Munir S, Zeb N, Ullah A, Khan B, Ali J (2019). Green nanotechnology: a review on green synthesis of silver nanoparticles—an ecofriendly approach. Int J Nanomedicine.

[3] Pallavi SS, Rudayni HA, Bepari A, Niazi SK, Nayaka S (2022). Green synthesis of Silver nanoparticles using *Streptomyces hirsutus* strain SNPGA-8 and their characterization, antimicrobial activity, and anticancer activity against human lung carcinoma cell line A549. Saudi J Biol Sci.

[4] Birla SS, Gaikwad SC, Gade AK, Rai MK (2013). Rapid synthesis of silver nanoparticles from *Fusarium oxysporum* by optimizing physicocultural conditions. Sci World J.

[5] Siddiqi KS, Husen A, Rao RAK (2018). A review on biosynthesis of silver nanoparticles and their biocidal properties. J Nanobiotechnology.

[6] Govindaraju K, Tamilselvan S, Kiruthiga V, Singaravelu G (2010). Biogenic silver nanoparticles by Solanum torvum and their promising antimicrobial activity. J Biopestic.

[7] Tian S, Saravanan K, Mothana RA, Ramachandran G, Rajivgandhi G, Manoharan N (2020). Anti-cancer activity of biosynthesized silver nanoparticles using *Avicennia marina* against A549 lung cancer cells through ROS/mitochondrial damages. Saudi J Biol Sci.

[8] Selvakumar P, Viveka S, Prakash S, Jasminebeaula S, Uloganathan R (2012). Antimicrobial activity of extracellularly synthesized silver nanoparticles from marine derived *Streptomyces rochei*. Int J Pharma Bio Sci.

[9] Lee SH, Jun BH (2019). Silver nanoparticles: synthesis and application for nanomedicine. Int J Mol Sci.

[10] Sung YK, Kim SW (2019). Recent advances in the development of gene delivery systems. Biomater Res.

[11] Zahin N, Anwar R, Tewari D, Kabir MT, Sajid A, Mathew B (2020). Nanoparticles and its biomedical applications in health and diseases: special focus on drug delivery. Environ Sci Pollut Res.

[12] Ahmed RH, Mustafa DE (2020). Green synthesis of silver nanoparticles mediated by traditionally used medicinal plants in Sudan. Int Nano Lett.

[13] Kaur A, Preet S, Kumar V, Kumar R, Kumar R (2019). Synergetic effect of vancomycin loaded silver nanoparticles for enhanced antibacterial activity. Colloids Surf B Biointerfaces.

[14] Feizi S, Taghipour E, Ghadam P, Mohammadi P (2018). Antifungal, antibacterial, antibiofilm and colorimetric sensing of toxic metals activities of eco friendly, economical synthesized Ag/AgCl nanoparticles using *Malva sylvestris* leaf extracts. Microb Pathog.

[15] Rajivgandhi G, Maruthupandy M, Quero F, Li WJ (2019). Graphene/nickel oxide nanocomposites against isolated ESBL producing bacteria and A549 cancer cells. Mater Sci Eng C.

[16] Dixit D, Gangadharan D, Popat KM, Reddy CRK, Trivedi M, Gadhavi DK (2018). Synthesis, characterization and application of green seaweed mediated silver nanoparticles (AgNPs) as antibacterial agents for water disinfection. Water Sci Technol.

[17] Suganya S, Ishwarya R, Jayakumar R, Govindarajan M, Alharbi NS, Kadaikunnan S (2019). New insecticides and antimicrobials derived from *Sargassum wightii* and *Halimeda gracillis* seaweeds: toxicity against mosquito vectors and antibiofilm activity against microbial pathogens. South African J Bot.

[18] Inbathamizh L, Ponnu TM, Mary EJ (2013). In vitro evaluation of antioxidant and anticancer potential of *Morinda pubescens* synthesized silver nanoparticles. J Pharm Res.

[19] Galvez AM, Ramos KM, Teja AJ, Baculi R (2019). Bacterial exopolysaccharide-mediated synthesis of silver nanoparticles and their application on bacterial biofilms. J Microbiol Biotechnol Food Sci.

[20] Haefeli C, Franklin C, Hardy K (1984). Plasmid-determined silver resistance in Pseudomonas stutzeri isolated from a silver mine. J Bacteriol.

[21] Thomas R, Janardhanan A, Varghese RT, Soniya EV, Mathew J, Radhakrishnan EK (2014). Antibacterial properties of silver nanoparticles synthesized by marine *Ochrobactrum *sp. Brazilian J Microbiol.

[22] Klaus T, Joerger R, Olsson E, Granqvist CG (1999). Silver-based crystalline nanoparticles, microbially fabricated. Proc Natl Acad Sci U S A.

[23] El-Dein MMN, Baka ZAM, Abou-Dobara MI, El-Sayed AKA, El-Zahed MM (2021). Extracellular biosynthesis, optimization, characterization and antimicrobial potential of escherichia coli D8 silver nanoparticles. J Microbiol Biotechnol Food Sci.

[24] Annamalai J, Nallamuthu T (2016). Green synthesis of silver nanoparticles: characterization and determination of antibacterial potency. Appl Nanosci.

[25] Quinteros MA, Aiassa Martínez IM, Dalmasso PR, Páez PL (2016). Silver nanoparticles: biosynthesis using an ATCC reference strain of pseudomonas aeruginosa and activity as broad spectrum clinical antibacterial agents. Int J Biomater.

[26] Paul M, Londhe VY (2019). Pongamia pinnata seed extract-mediated green synthesis of silver nanoparticles: preparation, formulation and evaluation of bactericidal and wound healing potential. Appl Organomet Chem.

[27] Bocate KP, Reis GF, de Souza PC, Oliveira Junior AG, Durán N, Nakazato G (2019). Antifungal activity of silver nanoparticles and simvastatin against toxigenic species of *Aspergillus*. Int J Food Microbiol.

[28] Khaleghi M, Madani M, Parsia P (2017). Biosynthesis characteristic of silver nanoparticles produced by mine soil bacteria isolation, Kerman, Iran. Int J Nanosci Nanotechnol.

[29] Kaliamurthi S, Selvaraj G, Çakmak ZE, Çakmak T (2016). Production and characterization of spherical thermostable silver nanoparticles from *Spirulina platensis* (Cyanophyceae). Phycologia.

[30] Pourali P, Yahyaei B (2016). Biological production of silver nanoparticles by soil isolated bacteria and preliminary study of their cytotoxicity and cutaneous wound healing efficiency in rat. J Trace Elem Med Biol.

[31] Kalimuthu K, Suresh Babu R, Venkataraman D, Bilal M, Gurunathan S (2008). Biosynthesis of silver nanocrystals by *Bacillus licheniformis*. Colloids Surf B Biointerfaces.

[32] Das VL, Thomas R, Varghese RT, Soniya EV, Mathew J, Radhakrishnan EK (2014). Extracellular synthesis of silver nanoparticles by the *Bacillus* strain CS 11 isolated from industrialized area. 3 Biotech.

[33] Quester K, Avalos-Borja M, Castro-Longoria E (2013). Biosynthesis and microscopic study of metallic nanoparticles. Micron.

[34] Elgorban AM, El-Samawaty AE-RM, Abd-Elkader OH, Yassin MA, Sayed SRM, Khan M (2017). Bioengineered silver nanoparticles using *Curvularia pallescens* and its fungicidal activity against *Cladosporium fulvum*. Saudi J Biol Sci.

[35] Lotfy WA, Alkersh BM, Sabry SA, Ghozlan HA (2021). Biosynthesis of silver nanoparticles by *Aspergillus terreus*: characterization, optimization, and biological activities. Front Bioeng Biotechnol.

[36] Abd-Elnaby HM, Abo-Elala GM, Abdel-Raouf UM, Hamed MM (2016). Antibacterial and anticancer activity of extracellular synthesized silver nanoparticles from marine *Streptomyces rochei* MHM13. Egypt J Aquat Res.

[37] Saravanan M, Vemu AK, Barik SK (2011). Rapid biosynthesis of silver nanoparticles from Bacillus megaterium (NCIM 2326) and their antibacterial activity on multi drug resistant clinical pathogens. Colloids Surf B Biointerfaces.

[38] Nayaka S, Chakraborty B, Bhat MP, Nagaraja SK, Airodagi D, Swamy PS (2020). Biosynthesis, characterization, and in vitro assessment on cytotoxicity of actinomycete-synthesized silver nanoparticles on Allium cepa root tip cells. Beni-Suef Univ J Basic Appl Sci.

[39] Sastry M, Patil V, Sainkar SR (1998). Electrostatically controlled diffusion of carboxylic acid derivatized silver colloidal particles in thermally evaporated fatty amine films. J Phys Chem B.

[40] Leclerc H (1962). Étude biochimique d’Enterobacteriaceae pigmentées. Ann Inst Pasteur (Paris).

[41] Tamura K, Sakazaki R, Kosako Y, Yoshizaki E (1986). Leclercia adecarboxylata Gen. Nov., Comb. Nov., formerly known as Escherichia adecarboxylata. Curr Microbiol.

[42] Mazzariol A, Zuliani J, Fontana R, Cornaglia G (2003). Isolation from blood culture of a Leclercia adecarboxylata strain producing an SHV-12 extended-spectrum beta-lactamase. J Clin Microbiol.

[43] Keyes J, Johnson EP, Epelman M, Cadilla A, Ali S. Leclercia adecarboxylata: an emerging pathogen among pediatric infections. Cureus. 2020. https://www.cureus.com/articles/29207-leclercia-adecarboxylata-an-emerging-pathogen-among-pediatric-infections 10.7759/cureus.8049 PMC7286590 32537268

[44] Javed B, Nadhman A, Mashwani ZUR (2020). Optimization, characterization and antimicrobial activity of silver nanoparticles against plant bacterial pathogens phyto-synthesized by *Mentha longifolia*. Mater Res Express.

[45] El-Saadony MT, El-Wafai NA, El-Fattah HIA, Mahgoub SA (2019). Biosynthesis, optimization and characterization of silver nanoparticles using a soil isolate of bacillus pseudomycoides MT32 and their antifungal activity against some pathogenic fungi. Adv Anim Vet Sci.

[46] El-Saadony MT, El-wafai NA, El-fattah HIA, Mahgoub SA (2018). Biosynthesis, optimization and characterization of silver nanoparticles biosynthesized by *Bacillus subtilis* ssp spizizenii MT5 isolated from heavy metals polluted soil. Zagazig J Agric Res.

[47] Thamilselvi V, Radha KV (2013). Synthesis of silver nanoparticles from *Pseudomonas putida* NCIM 2650 in silver nitrate supplemented growth medium and optimization using response surface methodology. Dig J Nanomater Biostructures.

[48] El-Batal AI, Amin MA, Shehata MMK, Hallol MMA (2013). Synthesis of silver nanoparticles by *Bacillus stearothermophilus* using gamma radiation and their antimicrobial activity. World Appl Sci J.

[49] Abo-State MAM, Partila AM (2015). Microbial production of silver nanoparticles by *Pseudomonas aeruginosa* cell free extract. J Ecol Heal Environ An Int J.

[50] Nindawat S, Agrawal V (2019). Fabrication of silver nanoparticles using Arnebia hispidissima (Lehm) A. DC. root extract and unravelling their potential biomedical applications. Artif Cells Nanomed Biotechnol.

[51] Christensen L, Vivekanandhan S, Misra M, Mohanty AK (2011). Biosynthesis of silver nanoparticles using *Murraya koenigii* (curry leaf): an investigation on the effect of broth concentration in reduction mechanism and particle size. Adv Mater Lett.

[52] Dwivedi AD, Gopal K (2010). Biosynthesis of silver and gold nanoparticles using Chenopodium album leaf extract. Colloids Surf A Physicochem Eng Asp.

[53] Saxena J, Sharma PK, Sharma MM, Singh A (2016). Process optimization for green synthesis of silver nanoparticles by *Sclerotinia sclerotiorum* MTCC 8785 and evaluation of its antibacterial properties. Springerplus.

[54] Saeed S, Iqbal A, Ashraf MA (2020). Bacterial-mediated synthesis of silver nanoparticles and their significant effect against pathogens. Environ Sci Pollut Res.

[55] Halima R, Archna A (2016). A review on green synthesis of silver nanoparticl, charactrization and optimization parameters. Int J Res Eng Technol.

[56] Balakumaran MD, Ramachandran R, Balashanmugam P, Mukeshkumar DJ, Kalaichelvan PT (2016). Mycosynthesis of silver and gold nanoparticles: optimization, characterization and antimicrobial activity against human pathogens. Microbiol Res.

[57] Saware K, Venkataraman A (2014). Biosynthesis and characterization of stable silver nanoparticles using *Ficus religiosa* leaf extract: a mechanism perspective. J Clust Sci.

[58] El-Rafie MH, Shaheen TI, Mohamed AA, Hebeish A (2012). Bio-synthesis and applications of silver nanoparticles onto cotton fabrics. Carbohydr Polym.

[59] Singh D, Rathod V, Ninganagouda S, Hiremath J, Singh AK, Mathew J (2014). Optimization and characterization of silver nanoparticle by endophytic Fungi *Penicillium* sp. Isolated from curcuma longa (turmeric) and application studies against MDR E. coli and *S. aureus*. Bioinorg Chem Appl.

[60] Krishnaraj C, Ramachandran R, Mohan K, Kalaichelvan PT (2012). Optimization for rapid synthesis of silver nanoparticles and its effect on phytopathogenic fungi. Spectrochim Acta Part A Mol Biomol Spectrosc.

[61] Ma L, Su W, Liu JX, Zeng XX, Huang Z, Li W (2017). Optimization for extracellular biosynthesis of silver nanoparticles by *Penicillium aculeatum* Su1 and their antimicrobial activity and cytotoxic effect compared with silver ions. Mater Sci Eng C.

[62] Nayak RR, Pradhan N, Behera D, Pradhan KM, Mishra S, Sukla LB (2011). Green synthesis of silver nanoparticle by *Penicillium purpurogenum* NPMF: the process and optimization. J Nanoparticle Res.

[63] Makarov VV, Love AJ, Sinitsyna OV, Makarova SS, Yaminsky IV, Taliansky ME (2014). “Green” nanotechnologies: synthesis of metal nanoparticles using plants. Acta Naturae.

[64] Verma A, Mehata MS (2016). Controllable synthesis of silver nanoparticles using neem leaves and their antimicrobial activity. J Radiat Res Appl Sci.

[65] Gurunathan S, Kalishwaralal K, Vaidyanathan R, Venkataraman D, Pandian SRK, Muniyandi J (2009). Biosynthesis, purification and characterization of silver nanoparticles using Escherichia coli. Colloids Surf B Biointerfaces.

[66] Banu A, Rathod V (2011). Synthesis and characterization of silver nanoparticles by Rhizopus stolonifer. Int J Biomed Adv Res.

[67] Sarsar V, Selwal MK, Selwal KK (2015). Biofabrication, characterization and antibacterial efficacy of extracellular silver nanoparticles using novel fungal strain of *Penicillium atramentosum* KM. J Saudi Chem Soc.

[68] Ghojavand S, Madani M, Karimi J (2020). Green synthesis, characterization and antifungal activity of silver nanoparticles using stems and flowers of felty germander. J Inorg Organomet Polym Mater.

[69] Thamilselvi V, Radha KV (2013). Comparative study for biosynthesis of silver nanoparticles from *Pseudomonas putida* NCIM 2650. Asian J Microbiol Biotechnol Environ Sci.

[70] Iqtedar M, Aslam M, Akhyar M, Shehzaad A, Abdullah R, Kaleem A (2019). Extracellular biosynthesis, characterization, optimization of silver nanoparticles (AgNPs) using Bacillus mojavensis BTCB15 and its antimicrobial activity against multidrug resistant pathogens. Prep Biochem Biotechnol.

[71] Khan N, Jameel J (2016). Optimization of reaction parameters for silver nanoparticles synthesis from *Fusarium oxysporum* and determination of silver nanoparticles concentration. J Mater Sci Eng.

[72] Mittal AK, Kaler A, Banerjee UC (2012). Free radical scavenging and antioxidant activity of silver nanoparticles synthesized from flower extract of *Rhododendron dauricum*. Nano Biomed Eng.

[73] Ibrahim S, Ahmad Z, Manzoor MZ, Mujahid M, Faheem Z, Adnan A (2021). Optimization for biogenic microbial synthesis of silver nanoparticles through response surface methodology, characterization, their antimicrobial, antioxidant, and catalytic potential. Sci Rep.

[74] Rose GK, Soni R, Rishi P, Soni SK (2019). Optimization of the biological synthesis of silver nanoparticles using *Penicillium oxalicum* GRS-1 and their antimicrobial effects against common food-borne pathogens. Green Process Synth.

[75] Khan T, Ali GS (2020). Variation in surface properties, metabolic capping, and antibacterial activity of biosynthesized silver nanoparticles: comparison of bio-fabrication potential in phytohormone-regulated cell cultures and naturally grown plants. RSC Adv Royal Soc Chem.

[76] Mondal AH, Yadav D, Mitra S, Mukhopadhyay K (2020). Biosynthesis of silver nanoparticles using culture supernatant of *Shewanella* sp. ARY1 and Their antibacterial activity. Int J Nanomed.

[77] Singh H, Du J, Singh P, Yi TH (2018). Extracellular synthesis of silver nanoparticles by *Pseudomonas* sp. THG-LS1.4 and their antimicrobial application. J Pharm Anal.

[78] Suresh AK, Pelletier DA, Wang W, Moon J-W, Gu B, Mortensen NP (2010). Silver nanocrystallites: biofabrication using shewanella oneidensis, and an evaluation of their comparative toxicity on gram-negative and gram-positive bacteria. Environ Sci Technol.

[79] Hamida RS, Abdelmeguid NE, Ali MA, Bin-Meferij MM, Khalil MI (2020). Synthesis of silver nanoparticles using a novel cyanobacteria *Desertifilum* sp. Extract: their antibacterial and cytotoxicity effects. Int J Nanomed.

[80] Kumar SA, Abyaneh MK, Gosavi SW, Kulkarni SK, Pasricha R, Ahmad A (2007). Nitrate reductase-mediated synthesis of silver nanoparticles from AgNO3. Biotechnol Lett.

[81] Li Y, Li CX, Lin W, Wang SS, Zhang WX, Jiang YM (2021). Full evaluation of assimilatory and dissimilatory nitrate reduction in a new denitrifying bacterium *Leclercia adecarboxylata* strain AS3–1: characterization and functional gene analysis. Environ Technol Innov.

[82] Aboelfetoh EF, El-Shenody RA, Ghobara MM (2017). Eco-friendly synthesis of silver nanoparticles using green algae (Caulerpa serrulata): reaction optimization, catalytic and antibacterial activities. Environ Monit Assess.

[83] Sheik GB, Abdel RAIA, Alzeyadi ZA, AlGhonaim MI (2019). Application of Plackett-Burman design for optimization of silver nanoparticles produced by Streptomyces sp. Int J Adv Biotechnol Res.

[84] El-Naggar MY, Ramadan W, El-Hamamsy RA (2017). The application of mediated biosynthesized green silver nanoparticles by *Streptomyces griseorubens* in water treatment. J Pure Appl Microbiol.

[85] Gangadharan D, Sivaramakrishnan S, Nampoothiri KM, Sukumaran RK, Pandey A (2008). Response surface methodology for the optimization of alpha amylase production by *Bacillus amyloliquefaciens*. Bioresour Technol.

[86] Aghaie E, Pazouki M, Hosseini MR, Ranjbar M, Ghavipanjeh F (2009). Response surface methodology (RSM) analysis of organic acid production for Kaolin beneficiation by *Aspergillus niger*. Chem Eng J.

[87] Trivedi P, Khandelwal M, Srivastava P (2014). Statistically optimized synthesis of silver nanocubes from peel extracts of citrus limetta and potential application in waste water treatment. J Microb Biochem Technol.

[88] Halima R, Narula A, Sravanthi RR (2021). Optimization of process parameters for the green synthesis of silver nanoparticles using Plackett-Burman and 3-level Box-Behnken design. J Huazhong Univ Sci Technol.

[89] Zhang X-F, Liu Z-G, Shen W, Gurunathan S (2016). Silver nanoparticles: synthesis, characterization, properties, applications, and therapeutic approaches. Int J Mol Sci.

[90] Abdelghany TM, Al-Rajhi AMH, Al Abboud MA, Alawlaqi MM, Ganash Magdah A, Helmy EAM (2018). Recent advances in green synthesis of silver nanoparticles and their applications: about future directions. A review. Bionanoscience BioNanoScience.

[91] Ulaeto SB, Mathew GM, Pancrecious JK, Nair JB, Rajan TPD, Maiti KK (2020). Biogenic Ag nanoparticles from neem extract: their structural evaluation and antimicrobial effects against *Pseudomonas nitroreducens* and *Aspergillus unguis* (NII 08123). ACS Biomater Sci Eng.

[92] Akter S, Huq MA (2020). Biologically rapid synthesis of silver nanoparticles by *Sphingobium* sp. MAH-11T and their antibacterial activity and mechanisms investigation against drug-resistant pathogenic microbes. Artif Cells, Nanomed Biotechnol.

[93] Abo-State MAM, Partila AM (2018). Production of silver nanoparticles (AgNPs) by certain bacterial strains and their characterization. Nov Res Microbiol J.

[94] Rauwel P, Küünal S, Ferdov S, Rauwel E (2015). A review on the green synthesis of silver nanoparticles and their morphologies studied via TEM. Adv Mater Sci Eng.

[95] González-Castillo J, Rodriguez E, Jimenez-Villar E, Rodríguez D, Salomon-García I, de Sá GF (2015). Synthesis of Ag@Silica nanoparticles by assisted laser ablation. Nanoscale Res Lett.

[96] Murthy HCA, Desalegn Zeleke T, Ravikumar CR, Anil Kumar MR, Nagaswarupa HP (2020). Electrochemical properties of biogenic silver nanoparticles synthesized using *Hagenia abyssinica* (Brace) JF. Gmel. medicinal plant leaf extract. Mater Res Express.

[97] Abdelmigid HM, Morsi MM, Hussien NA, Alyamani AA, Al Sufyani NM (2021). Comparative analysis of nanosilver particles synthesized by different approaches and their antimicrobial efficacy. J Nanomater.

[98] Bhuyar P, Rahim MHA, Sundararaju S, Ramaraj R, Maniam GP, Govindan N (2020). Synthesis of silver nanoparticles using marine macroalgae *Padina* sp. and its antibacterial activity towards pathogenic bacteria. Beni-Suef Univ J Basic Appl Sci.

[99] Javed B, Nadhman A, Mashwani ZR (2020). Phytosynthesis of Ag nanoparticles from *Mentha longifolia*: their structural evaluation and therapeutic potential against HCT116 colon cancer, Leishmanial and bacterial cells. Appl Nanosci.

[100] Pirtarighat S, Ghannadnia M, Baghshahi S (2017). Antimicrobial effects of green synthesized silver nanoparticles using *Melissa officinalis* grown under in vitro condition. Nanomed J.

[101] Mobaraki F, Momeni M, Jahromi M, Kasmaie FM, Barghbani M, Yazdi MET (2022). Apoptotic, antioxidant and cytotoxic properties of synthesized AgNPs using green tea against human testicular embryonic cancer stem cells. Process Biochem.

[102] Kumar V, Wadhwa R, Kumar N, Maurya PK (2019). A comparative study of chemically synthesized and Camellia sinensis leaf extract-mediated silver nanoparticles. 3 Biotech.

[103] Jo JH, Singh P, Kim YJ, Wang C, Mathiyalagan R, Jin CG (2016). Pseudomonas deceptionensis DC5-mediated synthesis of extracellular silver nanoparticles. Artif Cells, Nanomed Biotechnol.

[104] Bawskar M, Deshmukh S, Bansod S, Gade A, Rai M (2015). Comparative analysis of biosynthesised and chemosynthesised silver nanoparticles with special reference to their antibacterial activity against pathogens. IET Nanobiotechnol.

[105] Ghetas HA, Abdel-Razek N, Shakweer MS, Abotaleb MM, Ahamad Paray B, Ali S (2022). Antimicrobial activity of chemically and biologically synthesized silver nanoparticles against some fish pathogens. Saudi J Biol Sci.

[106] Neethu S, Midhun SJ, Sunil MA, Soumya S, Radhakrishnan EK, Jyothis M (2018). Efficient visible light induced synthesis of silver nanoparticles by Penicillium polonicum ARA 10 isolated from *Chetomorpha antennina* and its antibacterial efficacy against Salmonella enterica serovar Typhimurium. J Photochem Photobiol B Biol.

[107] Nayaka S, Chakraborty B, Pallavi S, Bhat MP, Shashiraj K, Ghasti B (2020). Synthesis of biogenic silver nanoparticles using Zanthoxylum rhetsa (Roxb.) dc seed coat extract as reducing agent and in-vitro assessment of anticancer effect on a549 lung cancer cell line. Int J Pharm Res.

[108] Loo YY, Rukayadi Y, Nor-Khaizura M-A-R, Kuan CH, Chieng BW, Nishibuchi M (2018). In vitro antimicrobial activity of green synthesized silver nanoparticles against selected gram-negative foodborne pathogens. Front Microbiol.

[109] He Y, Ingudam S, Reed S, Gehring A, Strobaugh TP, Irwin P (2016). Study on the mechanism of antibacterial action of magnesium oxide nanoparticles against foodborne pathogens. J Nanobiotechnol BioMed Central.

[110] Bhat M, Chakraborty B, Kumar RS, Almansour AI, Arumugam N, Kotresha D (2021). Biogenic synthesis, characterization and antimicrobial activity of Ixora brachypoda (DC) leaf extract mediated silver nanoparticles. J King Saud Univ Sci.

[111] Gudikandula K, Charya MS (2016). Synthesis of silver nanoparticles by chemical and biological methods and their antimicrobial properties. J Exp Nanosci.

[112] Le Ouay B, Stellacci F (2015). Antibacterial activity of silver nanoparticles: a surface science insight. Nano Today.

[113] Yazdi MET, Khara J, Housaindokht MR, Sadeghnia HR, Bahabadi SE, Amiri MS (2019). Role of Ribes khorassanicum in the biosynthesis of AgNPs and their antibacterial properties. IET Nanobiotechnol.

[114] Taghavizadeh Yazdi ME, Darroudi M, Amiri MS, Zarrinfar H, Hosseini HA, Mashreghi M (2022). Antimycobacterial, anticancer, antioxidant and photocatalytic activity of biosynthesized silver nanoparticles using *Berberis Integerrima*. Iran J Sci Technol Trans A Sci.

[115] Rambabu K, Bharath G, Banat F, Show PL (2021). Green synthesis of zinc oxide nanoparticles using Phoenix dactylifera waste as bioreductant for effective dye degradation and antibacterial performance in wastewater treatment. J Hazard Mater.

[116] Rad SS, Sani AM, Mohseni S (2019). Biosynthesis, characterization and antimicrobial activities of zinc oxide nanoparticles from leaf extract of Mentha pulegium (L.). Microb Pathog.

[117] Mousavi-Kouhi SM, Beyk-Khormizi A, Amiri MS, Mashreghi M, Taghavizadeh Yazdi ME (2021). Silver-zinc oxide nanocomposite: from synthesis to antimicrobial and anticancer properties. Ceram Int.

[118] Zhang L, Jiang Y, Ding Y, Povey M, York D (2007). Investigation into the antibacterial behaviour of suspensions of ZnO nanoparticles (ZnO nanofluids). J Nanoparticle Res.

[119] Ganesh Babu MM, Gunasekaran P (2009). Production and structural characterization of crystalline silver nanoparticles from *Bacillus cereus* isolate. Colloids Surf B Biointerfaces.

[120] Kamusoko R, Jingura RM, Parawira W, Chikwambi Z, Basu C (2021). Purification and amplification of DNA from cellulolytic bacteria application for production from crop residues. Biofuels biodiesel methods mol biol.

[121] Tamura K, Stecher G, Kumar S (2021). MEGA11: molecular evolutionary genetics analysis version 11. Mol Biol Evol.

[122] Sarsar V, Selwal MK, Selwal KK (2014). Significant parameter in the optimization of biosynthesis of silver nanoparticles using *Psidium Guajava* leaf extract and evaluation of their antimicrobial activity against human pathogenic bacteria. Int J Adv Pharm Sci.

[123] Devi A, Sharma A, Sharma H, Sharma S (2017). In vitro biosynthesis and characterization of biosilver nanoparticles of *Pseudomonas putida* LUA 15. 1 and their potential as antibacterial agents. Int J Chem Stud.

[124] Plackett RL, Burman JP (1946). The design of optimum multifactorial experiments. Biometrika.

[125] Al-Sarrani AQM, El-Naggar MYM (2006). Application of Plackett-Burman factorial design to improve citrinin production in Monascus ruber batch cultures. Bot Stud.

[126] El-Naggar NE-A, Mohamedin A, Hamza SS, Sherief A-D (2016). Extracellular biofabrication, characterization, and antimicrobial efficacy of silver nanoparticles loaded on cotton fabrics using newly isolated Streptomyces sp. SSHH-1E. J Nanomater.

[127] Mahmoud MA, Al-Sohaibani SA, Al-Othman MR, Abd El-Aziz AM, Eifan SA (2013). Synthesis of extracellular silver nanoparticles using *Fusarium semitectum* (KSU-4) isolated from Saudi Arabia. Dig J Nanomater Biostruct.

[128] Buszewski B, Railean-Plugaru V, Pomastowski P, Rafińska K, Szultka-Mlynska M, Golinska P (2018). Antimicrobial activity of biosilver nanoparticles produced by a novel Streptacidiphilus durhamensis strain. J Microbiol Immunol Infect.

[129] Fahmy A, Kamoun EA, El-Eisawy R, El-Fakharany EM, Taha TH, El-Damhougy BK (2015). Poly(vinyl alcohol)-hyaluronic acid membranes for wound dressing applications: synthesis and in vitro bio-evaluations. J Braz Chem Soc.

